# Smart Silk Origami as Eco-sensors for Environmental Pollution

**DOI:** 10.1021/acsabm.2c00023

**Published:** 2022-05-16

**Authors:** Saphia A. L. Matthew, Gemma Egan, Kimia Witte, Jirada Kaewchuchuen, Suttinee Phuagkhaopong, John D. Totten, F. Philipp Seib

**Affiliations:** † Strathclyde Institute of Pharmacy and Biomedical Sciences, University of Strathclyde, 161 Cathedral Street, GlasgowG4 0RE, U.K.; § EPSRC Future Manufacturing Research Hub for Continuous Manufacturing and Advanced Crystallisation (CMAC), University of Strathclyde, Technology and Innovation Centre, 99 George Street, GlasgowG1 1RD, U.K.

**Keywords:** silk fibroin, semi-autonomy, reusable material, origami, pollution sensor

## Abstract

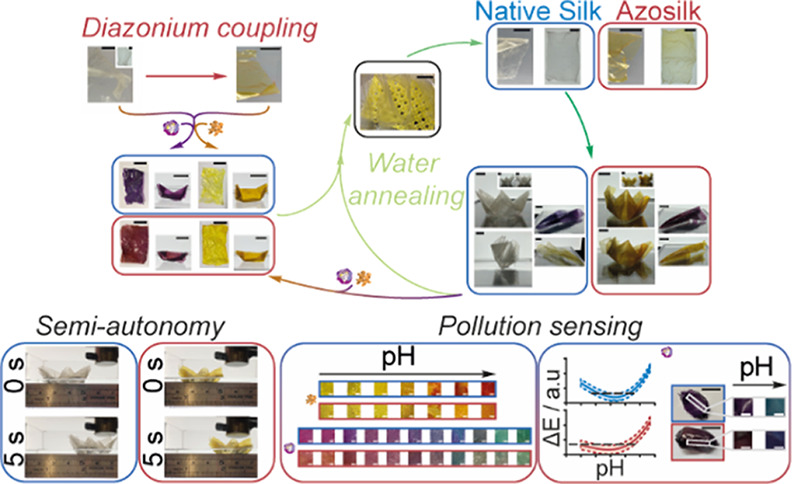

Origami folding is an easy, cost-effective, and scalable fabrication method for changing a flat material into a complex 3D functional shape. Here, we created semicrystalline silk films doped with iron oxide particles by mold casting and annealing. The flat silk films could be loaded with natural dyes and folded into 3D geometries using origami principles following plasticization. They performed locomotion under a magnetic field, were reusable, and displayed colorimetric stability. The critical parameters for the design of the semi-autonomous silk film, including ease of folding, shape preservation, and locomotion in the presence of a magnetic field, were characterized, and pH detection was achieved by eye and by digital image colorimetry with a response time below 1 min. We demonstrate a practical application—a battery-free origami silk boat—as a colorimetric sensor for waterborne pollutants, which was reusable at least five times. This work introduces silk eco-sensors and merges responsive actuation and origami techniques.

## Introduction

The craftsman art of origami has been used for over four centuries to change a flat material into a complex 3D shape.^1^ Folding is an easy, cost-effective, and scalable fabrication method;^1−4^ therefore, origami has inspired a variety of structures over a wide size range, from DNA-origami^5^ and soft robotics^4,6^ to meter-scale shelters.^7^ As origami is a compliant mechanism that requires the deformation of elastic members,^3^ tailoring the stiffness and softness of the flat material is important for obtaining a compliant, foldable architecture, which preserves its final shape.^8^ The origami folds can endow the structure with attractive mechanical properties, such as load bearing capacity^1,9^ and impact absorption;^10−12^ consequently, folds are being increasingly incorporated into deployable modules.^2,4,8,13^

Environmentally responsive actuators, which can convert external physical forces to mechanical force, have attracted growing interest for a diverse range of applications, including drug delivery,^14,15^ biomedical devices,^14−16^ and sensors.^16−18^ Examples of external stimuli used as energy sources for locomotion include thermal energy,^19^ humidity,^17,20−23^ chemicals,^18^ and optical^19,24^ and magnetic fields.^25^ Among these sources, magnetic fields typically result in rapid and directional actuation over long ranges. Materials for smart actuators range from graphene^26^ and metal–organic frameworks^27^ to synthetic polymers.^28^ However, these materials can raise environmental sustainability issues and require harsh, multi-step reaction conditions for production; therefore, emphasis is shifting to bioresorbable metals^29^ and ecofriendly polymers^18,21,30,31^ that are naturally sourced, renewable, and biodegradable. The ecological footprint of waterborne devices is particularly important, as freshwater and marine environments are already negatively impacted by plastic and chemical pollution.^32^

A global need exists for *in situ* water quality monitoring of large or complex water distribution systems and wastewater effluents to mitigate the impact of environmental contaminants on health.^33−35^ Miniaturized optical,^34^ electrical,^34,36^ magnetic,^34^ and chemical sensors^34,36−38^ capable of on-site detection provide a promising alternative to slow traditional analytical methods. These technologies should be inherently green themselves, so interest is growing in developing nontoxic, natural colorimetric indicators loaded within a biopolymer matrix as eco-green chemical sensors. For example, curcuminoids from turmeric and anthocyanins from red-pigmented plants are metal and pH-responsive dyes^39,40^ and have been used with biodegradable polymers including chitosan^41,42^ and corn and tapioca starch^31,39,43^ as visual pH-sensing films. However, at present, no silk fibroin matrices or complete examples of portable eco-green sensors have been reported. The folding of biocompatible, biodegradable, and sustainable^44^ silk fibroin films into reusable origami devices could serve as a simple approach for the fabrication of ecofriendly early warning systems for waterborne pollutants.

## Results and Discussion

**Figure 1:**
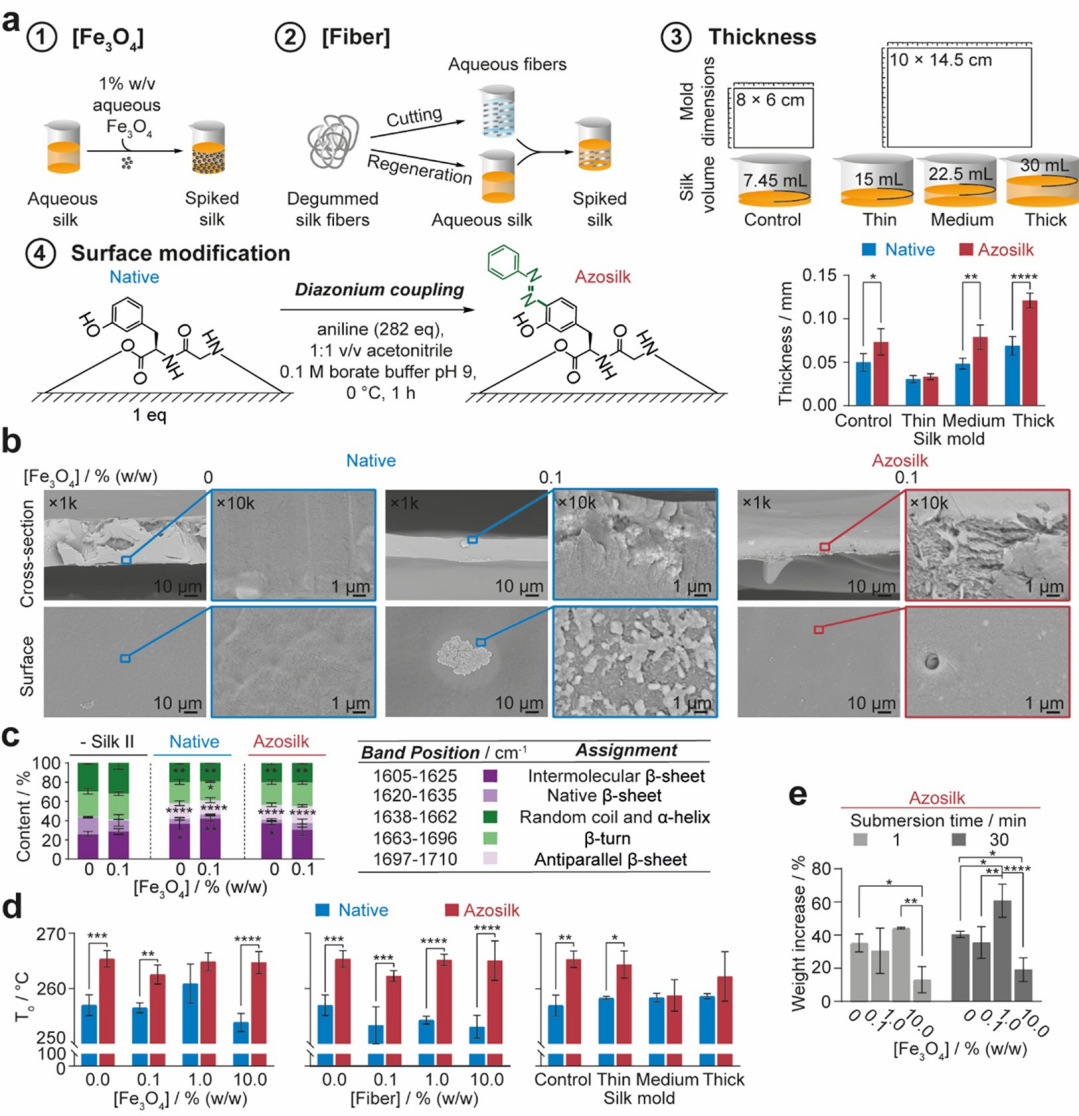
Properties of drop-casted silk films varying in diazonium coupling, fiber content, iron oxide content, and thickness. (a) Factors investigated during the preparation of silk films and the thickness of silk films obtained by varying mold size and silk solution volume during casting. (b) SEM images of native silk and azosilk films after water annealing with 0 and 0.1% (w/w) iron oxide particle loads. (c) FTIR band assignments and schematic key. Secondary structure content of silk films drop-casted with varying iron oxide particle concentrations. Secondary structure content (%) was calculated from the relative areas of peaks in the second-derivative spectrum. Untreated silk films were used as negative controls for β-sheet content. The secondary structure contents of multiple factors were evaluated by ordinary one-way analysis of variance (ANOVA), followed by Dunnett’s multiple comparison, post-hoc test against the secondary structure content of the native, negative silk II control containing 0% (w/w) iron oxide. (d) Extrapolated onset temperature of decomposition (*T*_o_) of silk films from first-cycle DSC. (e) Swelling of silk films with varying iron oxide particle concentrations following immersion in water. (e) Error bars are hidden in the bars and plot symbols when not visible, ± SD, *n* = 3. Multiple factors were evaluated by two-way analysis of variance (ANOVA), followed by Šidák’s multiple comparison, simple effects post-hoc test. Asterisks denote statistical significance determined using post-hoc tests as follows: *p < 0.05, **p < 0.01, ***p < 0.001, ****p < 0.0001.

**Figure 2:**
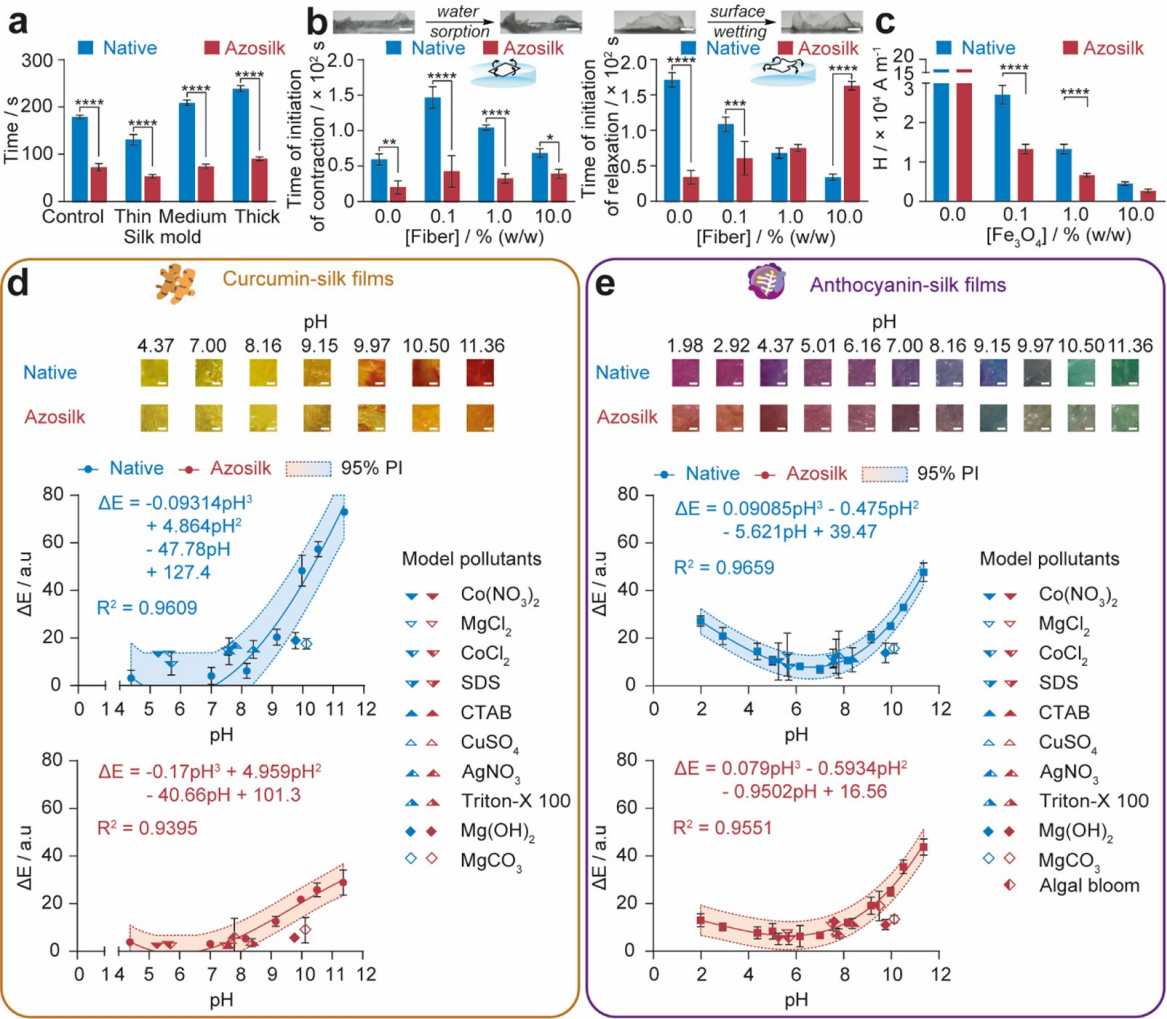
Mold-casted native silk and azosilk films demonstrate semi-autonomy and sensing capability. (a) Time window for folding silk films while plasticized following water annealing. (b) Time taken for initiation of contraction and initiation of relaxation by films lying on the air–water interface was used as a measure of structural stability in wet environments. (c) Electromagnetic field strength (H) as a function of the distance from the surface of an N52 rare-earth neodymium round cylinder magnet that was able to pull a floating rectangular-shaped silk film (0.1 g, 25 μm) loaded with iron oxide particles along the water–air interface. The correlation between the environmental pH and color change (ΔE) of (d) curcumin-loaded and (e) anthocyanin-loaded silk films, where ΔE was measured with the mean intensities in the Lab color space. The predictive accuracy of the cubic polynomial equations was evaluated using the 95% prediction interval and the measured ΔE of films treated with randomly grab sampled algal blooms and environmentally relevant concentrations of surfactants and heavy metal complexes, which served as model pollutants. ± SD, *n* = 3. Error bars are hidden in the bars and plot symbols when not visible. Scale bars = 0.5 cm. Multiple factors were evaluated by two-way analysis of variance (ANOVA), followed by Šidák’s multiple comparison, simple effects post-hoc test. Asterisks denote statistical significance determined using the post-hoc tests as follows: *p < 0.05, **p < 0.01, ***p < 0.001, ****p < 0.0001.

**Figure 3:**
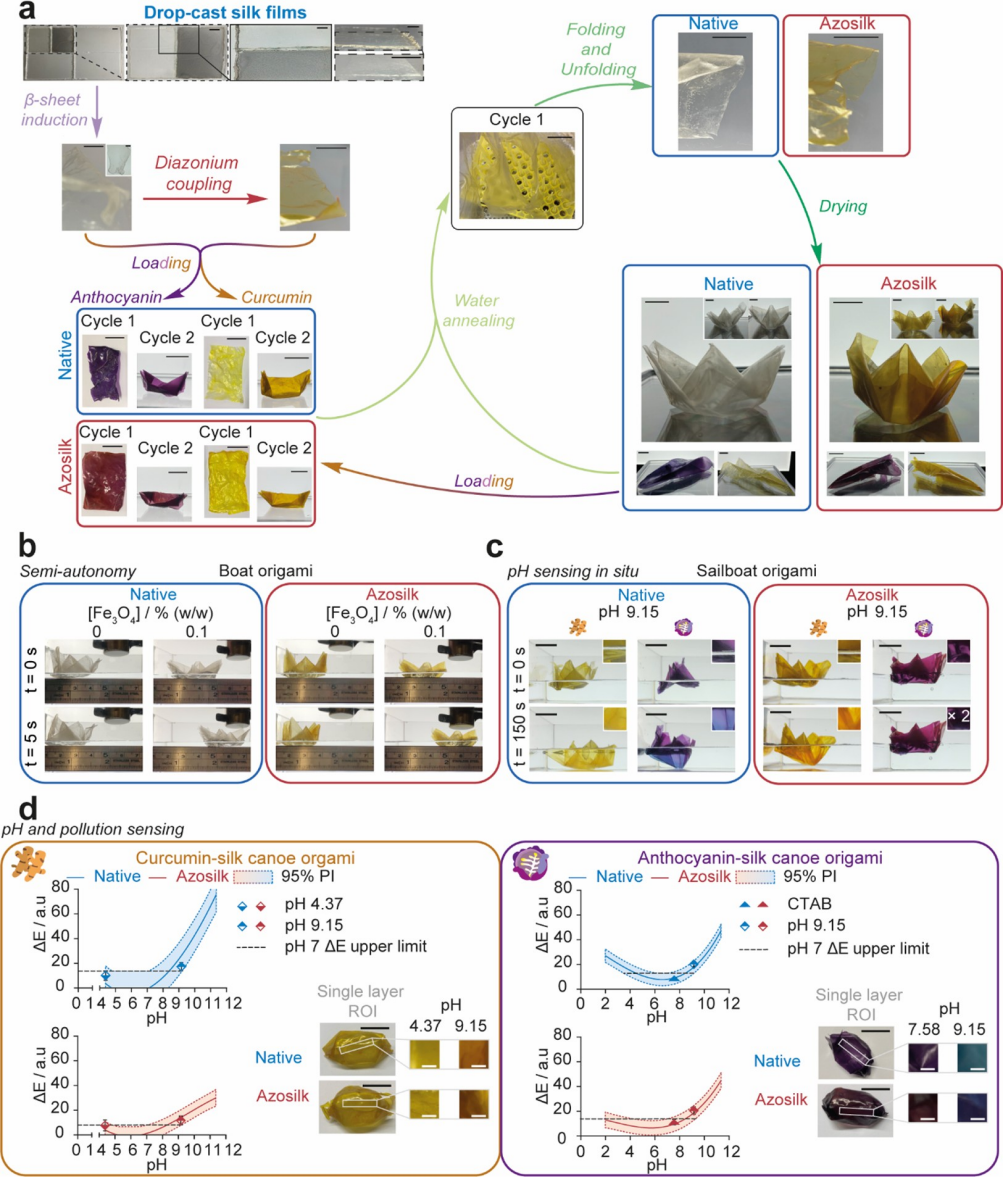
Smart silk origami as a structurally stable environmental pollution sensor. (a) Simplified origami workflow for the preparation of 3D, reusable devices. For the complete workflow, refer to Figure S13. (b) Semi-autonomous movement of origami silk boats across water using a magnet. (c) Visual detection of color changes in silk sailboat origami with pH. (d) Sensing capability of silk canoe origami to pH and contaminated environmental water models using the 95% prediction interval of the cubic polynomial calibration curves for ΔE and pH. ± SD, *n* = 3. Error bars are hidden in the bars and plot symbols when not visible. Scale bars = 2 cm. Asterisks denote statistical significance determined using the *t*-test and post-hoc tests as follows: *p < 0.05, **p < 0.01, ***p < 0.001, ****p < 0.0001.

Here, 3D silk structures were folded *via* cast molding of liquid silk and plasticization of the 2D film through water annealing (Figure 1). Liquid silk was also spiked with iron oxide particles to realize semi-autonomous films, which displayed a magnetic response in the presence of an electromagnetic field with a field strength (H) of 0.27 × 10^4^ A m^–1^, at an iron oxide doping concentration of 0.1% (w/w) of silk protein and 0.1 g silk film mass (Figure 2). The surface of water-insoluble films was also modified by diazonium coupling with benzene diazonium to increase hydrophobic repulsive forces (Figure 1), and this, in turn, increased the buoyancy and magnetically driven actuation of the resulting azosilk films. Both native and azosilk films could be loaded with curcumin and anthocyanin to fabricate colorimetric 3D silk boats for detection of heavy metal salts, surfactants, and algae at harmful aqueous concentrations (Figures 2 and 3), thereby demonstrating their potential in pollution sensing applications.

Casting conditions were first optimized for semi-autonomy by varying the iron oxide particle concentration, the time window for folding by tuning film thickness, and the longevity of folds upon water by tuning surface hydrophobicity and silk microfiber concentration (Figure 1a). The silk microfiber content was varied by doping silk feeds with a batch of silk microfibers of 1241.9 ± 790.4 μm in length and 21.0 ± 2.9 μm in width (Figure S1), previously manufactured by mechanically shearing degummed silk fibers. The film thickness was tuned by increasing the volume of liquid silk for mold casting. The crystallinity of silk fibroin films can increase with drying time;^44^ therefore, the films were methanol annealed to induce β-sheet self-assembly for scalable origami production and to enable the loading of lipophilic dyes during this step. Water annealing was used to increase the plasticity and fracture resistance of the semicrystalline films^45,46^ during folding. The water molecules permeate the silk network and are involved in silk-water hydrogen bonding, which causes structural reorganization, primarily in the amorphous regions of high α-helix and random coil secondary structure content.^45^ This process significantly increases silk chain mobility, while stiffness is regained upon drying. Scanning electron microscopy (SEM) confirmed that iron oxide particles were incorporated into, and retained within, the silk film matrix following post-processing methods (Figure 1b). The post-casting modification of silk films by diazonium coupling^47,48^ of benzene diazonium with tyrosine and histidine residues was used to increase the hydrophobicity of the film surface. Diazonium coupling generally increased the film thickness compared to native films (Figure 1a) and resulted in an increase in the water contact angle, indicating a greater hydrophobicity (Figure S2a,b).

All films showed stability in water due to the dominant β-sheet secondary structure, which ranged from 55 to 61% (Figure 1c). The thermal decomposition of silk films occurred at temperatures above 250 °C, which was consistent with a high β-sheet content,^44^ and azo-modification generally increased the thermal stability (Figure 1d, Tables S1 and S2, and Figures S3 and S4). Stability at high temperature is advantageous as it increases the lifespan of the silk films across a range of atmospheric environments. A low degree of swelling in water is also desirable to preserve the stiffness and shape of the 3D architecture. All films showed a low degree of swelling in water within 30 min, ranging from 19 to 61%, although water uptake was most significant within 5 min and leveled off thereafter (Figure 1e and Figure S2c). Weight change was not affected by the thickness or iron oxide loads of native silk films, although the water uptake over 30 min was maximal for silk films spiked with 0.1% (w/w) fiber at 46%. Azosilk films showed varying swelling over 30 min with iron oxide particle doping. For example, a weight change of 35% occurred at an iron oxide loading of 0.1% (w/w), and this increased to 61% at 1% (w/w) iron oxide. Increasing the iron oxide content further to 10% (w/w) resulted in a lower weight increase of 19% over 30 min.

The silk film properties, which impacted the deployment and lifetime of the origami architecture, were optimized by varying the silk fiber and iron oxide content and the film thickness (Figure 2a–c). Following plasticization by water annealing, the time of plastic endurance of the azosilk and native silk films in air increased with thickness due to the reduced surface area percentage for evaporation of the thicker films (Figure 2a). Across all thicknesses, azo-modification reduced the plasticity time window (Figure 2a). We hypothesized that increasing the silk fiber content could increase silk film stiffness, reduce water sorption, and prolong the architecture lifespan. The edges of the films were found to wrinkle and curl when floated on water (Figure 2b), a contraction process likely caused by a water sorption gradient. Relaxation of the films then occurred, and the films reassumed their original shape, possibly due to surface wetting (Figure 2b). The time taken for initiation of contraction and for initiation of relaxation by films when floating on the air-water interface was used as a measure of shape preservation. For the native silk films, increasing the silk fiber loading from 0 to 10% caused faster relaxation upon contact with water, while this trend was reversed by azo-modification (Figure 2b). Consequently, azosilk films with silk fiber contents below 10% (w/w) exhibited faster relaxation than native silk films. Finally, all films spiked with iron oxide showed semi-autonomy on water, as they could be pulled along the air–water interface with a cylindrical neodymium magnet (Figure S2d). Increasing the iron oxide load from 0.1 to 10% (w/w) resulted in decreased electromagnetic field strengths (H) required for magnetic response, from 2.71 × 10^4^ to 0.45 × 10^4^ A m^–1^ for native films and from 1.33 × 10^4^ to 0.27 × 10^4^ A m^–1^ for azosilk films (Figure 2c). We speculate that for equivalent iron oxide loadings and thereby equivalent magnetic susceptibilities, the azosilk films show semi-autonomy at longer distances compared to the native films due to reduced viscous drag for azosilk films than for native silk films. The azosilk films glide better on the water surface as the diazonium coupling increased the presence of defects and air pockets on the film surface (Figure 1b) and increased hydrophobic repulsion with the water interface (Figure S2a). Due to the rough surface of the films, further experimentation is required to identify the reduction in viscous drag caused by the increased hydrophobicity of the azosilk films.

Quantification of the color change of anthocyanin and curcumin-loaded polymer films and fibers in response to pH is an active research area for the detection of ammonia produced during food spoilage.^49^ Digital image colorimetry, combined with principle component analysis^39^ and non-linear regression,^50^ has been successfully utilized in these smart packaging systems to enable recognition of spoiled foodstuffs^39^ and ammonia content^50^ from the degree of the color change exhibited. However, to the best of our knowledge, we provide the first example of silk fibroin-based, eco-sensors for the detection of waterborne, environmental pollutants using curcumin and anthocyanin as natural, nontoxic pH indicators.

For sensing applications, silk films doped with 0.1% (w/w) iron oxide particles and casted at medium thickness were loaded with curcumin extracted from turmeric rhizome and anthocyanin extracted from fresh red cabbage (Figure S5). Both native and azosilk films showed a visible color change upon loading with the natural pigments (Figure S6) and could be used as colorimetric probes for pH changes (Figure 2d,e and Figure S7). The colors of the azosilk and native curcumin-loaded films were yellow between pH 4.37 and pH 8.16, yellow-orange between pH values of 9.15 and 10.50, and deep orange-red at pH 11.36 (Figure 2d and Figure S7). The color change confirmed the reversible tautomerization of curcumin from the predominant yellow keto form in the acidic and neutral environments to the red enol form as basicity increased above pH 8 (Figure S8a).^39^ The color change was analyzed in the RGB color space and revealed a reduction in the green channel intensity for native and azosilk films with increasing pH above pH 8.16, in addition to a reduction in the red channel intensity of native silk films above pH 10.59 (Figure S7).

Anthocyanin-loaded films were magenta-pink in acidic media below pH 4.37 due to the dominant cationic flavylium species^39,51^ and purple between pH 4.37 and pH 7.00. As equilibrium favored the quinoidal anhydrobase in alkaline media, the color of the films changed from purple-blue between pH 7.00 and pH 9.15 to blue-green between pH 9.15 and pH 11.36 (Figure 2e and Figures S7 and S8b).^39,51^ In the RGB color space, the green channel intensity of native films increased between pH 9.15 and pH 9.97, while the red channel intensity decreased for native and azosilk films as pH was raised from 1.98 to 4.37 and from 9.97 to 11.36 (Figure S7). Native silk films were more sensitive to pH than the azosilk films when loaded with curcumin or anthocyanin, as the relative color change was consistently greater than their azosilk counterparts across the pH range investigated (Figure S9a).

Calibration curves were constructed for the color change (ΔE) of silk films at pH values between 4.37 and 11.36 for curcumin-loaded films and pH values between 1.98 and 11.36 for anthocyanin-loaded films (Figure 2d–e). The ΔE values of curcumin and anthocyanin-loaded films displayed a positive correlation as pH increased from 7.00 to 11.36. Also, the ΔE values for anthocyanin-loaded films showed a negative correlation as pH increased from 1.98 to 7.00. For curcumin and anthocyanin-loaded films, cubic polynomial equations were fitted to ΔE and pH at *R*^2^ values above 0.93. The measured ΔE of all films treated with model pollutants of domestic, industrial, and municipal wastewater examples^52^ in addition to randomly grab sampled algal blooms lay within the 95% prediction intervals of the calibration curves.

Although curcumin and anthocyanin can coordinate with some heavy metals, namely, di- and trivalent metals (Figure S8),^31,51,53,54^ no measurable color changes were identified for the loaded native or azosilk films with 0.1% (w/w) iron oxide dopant at the lower limits^55−58^ of toxic concentrations of heavy metals at pH values below 9.76 (Figure 2d,e and Figures S9a and S10a). However, following exposure to Mg(OH)_2_ (pH 9.76) and MgCO_3_ (pH 10.12), the ΔE for curcumin and anthocyanin-loaded native and azosilk films was outside the 95% prediction intervals at the respective pH values (Figure 2d,e). This could indicate value as a novel sensing assay for the concentration of divalent heavy metals, provided that pH was controlled. Curcumin-silk films could be unloaded by treatment with sodium hydroxide,^59^ allowing recycling of the film and loading with alternative indicators (Figure 3 and Figure S6). However, unloading the azosilk films increased the film brittleness and led to fracture. This effect was likely a byproduct of polymer backbone scission and the resulting generation and growth of voids, cavities, and cracks.

The color change of the curcumin-azosilk, anthocyanin-azosilk, and anthocyanin-silk films following exposure to 0.2 M potassium phosphate buffer (pH 9.15) was stable following storage at 4 and 20 °C *in vacuo* for 31 days (Figures S9b and S11). Conversely, the curcumin-silk film only showed color stability when stored for 31 days at 4 °C (Figure S11). This stability was evidenced by the ΔE values for all film and indicator types following storage lying within the 95% prediction intervals of the calibration curves for the fresh films (Figure S11b). The mean pixel color intensity changes of all films in the RGB and Lab color spaces were determined directly using the smartphone application, ColorAssist Lite (Figure S11a,). The relative color changes of the azosilk and native silk films were not significantly different when measured directly using the smartphone application, and this reflected the results from digital postprocessing and segmentation using ImageJ (Figure S9b). Agreement between the 95% prediction intervals and measured ΔE using ColorAssist Lite was obtained for curcumin-azosilk films stored at 4 and 20 °C, curcumin-native silk films stored at 4 °C, anthocyanin-native silk stored at 20 °C, and anthocyanin-azosilk films stored at 20 °C (Figure S11b). Consequently, the omission of the digital postprocessing step would be expected to lower the accuracy of pH prediction. It is likely that this could be improved by constructing the calibration curves with data acquired directly in ColorAssist Lite, although the greater random error could result in larger prediction intervals and reduced sensitivity to drift in environmental pH. Nevertheless, the capability of digital image colorimetry using smartphone applications can streamline image analysis in field conditions by removing the image postprocessing and segmentation steps.^60^

As a proof of principle, native silk and azosilk medium thickness films containing 0.1% (w/w) iron oxide were then folded into a variety of 3D origami structures, including waterborne boats and airborne darts and spinners (Figure 3 and Figures S12 and S13).

Silk origami structures could be reused for at least five cycles prior to elastic failure upon folding, and the films could also be reloaded with chemical indicators (Figure 3a). We speculate that the simple, manual origami manufacturing process will aid the recycling of eco-sensors in remote locations and communities. In addition, both native and azosilk examples of 3D silk origami sailboats remained structurally stable for at least 3 days on ultrapure water. An efficient method for device distribution and recovery using magnetic fields as the energy source for locomotion was achieved, as the silk origami retained semi-autonomy upon folding (Figure 3b). Silk origami sailboats were then used as *in situ* pH probes and showed visible color changes along their hulls within 2.5 min of exposure to a 0.2 M potassium phosphate buffer at pH 9.15 (Figure 3c).

The silk canoe design enabled colorimetric analysis of the keel of the boat, as this region of interest consisted of a single layer. The color changes as pH increased from 4.37 to 9.15 for curcumin-loaded canoes and from pH values of 7.58 to 9.15 for anthocyanin-loaded canoes could be modeled using the 95% prediction intervals of the cubic polynomial correlation between ΔE and pH for the unfolded silk films (Figure 3 and Figures S9c and S10b). Both native silk and azosilk films loaded with curcumin or anthocyanin could be used for the detection of polluted aqueous environments at alkaline pH. The pH at which the upper ΔE limit and the lower ΔE limit of the 95% prediction intervals intersected was used to identify the minimum pH change from neutrality, which would be required to confirm polluted water. For example, for aqueous environments with a desired pH value of 7.00, the origami canoes could detect contamination with 95% accuracy when pH rises above pH 9. Additionally, increased aqueous acidity could also be probed with 95% accuracy using the anthocyanin-loaded, native silk origami canoes at pH values below 3. Consequently, the anthocyanin-loaded, native silk origami canoes provide the most suitable eco-sensors for the detection of contaminants ranging from excessive algae growth^61^ to acid rain.^62^ The simple image acquisition process means that data from deployed silk origami eco-sensors could be monitored in near real-time using field conditions or remotely by aerially acquired photography.

## Conclusions

In conclusion, soft origami devices were folded from silk fibroin films and loaded with natural, nontoxic dyes for colorimetric determination of environmental pH. The boats preserved their shape for at least 3 days on water and could exhibit color changes within 1 min after exposure to solutions of basic metal salts, surfactants, and algal blooms. These eco-green sensors demonstrate the practical importance of origami for engineering silk devices and enable a simple, deployable approach for direct monitoring of pH and pollution.

## Experimental Methods

### Materials

Studies were undertaken at 18–22 °C and reagents were obtained from Sigma Aldrich at purities of ≥98%, unless otherwise stated. Dialysis of silk fibroin was conducted using Slide-A-Lyzer dialysis cassettes (molecular weight cut-off 3500 g mol^–1^, Thermo Fisher Scientific Inc., Waltham, MA, USA). Polydimethylsiloxane (PDMS) for cast molding, HPLC-grade methanol, HPLC-grade acetonitrile, and anhydrous sodium carbonate (certified AR for analysis) were acquired from Fisher Chemical. Aniline (99.8%), *p*-toluenesulfonic acid (99%), and anhydrous lithium bromide (99%) were obtained from Acros Organics. Curcumin (95% total curcuminoid content from turmeric rhizome) was purchased from Alfa Aesar. Sodium nitrite (99%) was acquired from AnalaR NORMAPUR. Boric acid (99.5% ACS reagent) and sodium tetraborate decahydrate (99.5% ACS reagent) were obtained from Sigma Aldrich. All solvents and reagents were used without additional purification.

### Reverse Engineering of *Bombyx mori* Silk Cocoons

*B. mori* cocoons were degummed by boiling in 0.2 M aqueous Na_2_CO_3_ for 0.5 h and degummed silk fibroin dissolved in 9.3 M aqueous LiBr solution at 60 °C for 3 h, as described elsewhere.^63^ The regenerated silk solution was stored at 4 °C until use.

### Fabrication and Water Annealing of Silk Films

Liquid silk was mixed with a 10% w/v aqueous suspension of silk fiber (1241.9 ± 790.4 μm × 21.0 ± 2.9 μm) or 1% w/v iron(III) oxide (synthetic spherical particles with 99.995% < 325 mesh [∼45 μm] size, > 96.8% purity, 4.6 g/cm^3^ solid density and 0.8–1.2 g/cm^3^ bulk density from Inoxia Ltd., Sweden) to give a final suspension of 3% w/v silk containing 0.1, 1.0 or 10.0% dopant weight per silk weight. The liquid silk and silk suspensions were mixed slowly before casting in silicone molds (Sika Everbuild Building Products Ltd., Leeds, UK) on a Perspex base (RuudraScott Plastic, Glasgow, UK) in air for 16 h.

Four molds and silk volumes were used in the study. The control mold (8 × 6 cm; 7.45 mL) was used to screen iron oxide and silk fiber loads and to cast 3% w/v silk with 0.1% (w/w) iron oxide for loading with natural indicators. The film thickness was screened by increasing the casting volume in a 10 × 14.5 cm mold from 15 mL for thin films to 22.5 mL for medium films and 30 mL for thick films. The medium thickness mold was used to cast 3% w/v silk with 0.1% (w/w) iron oxide for origami. Films were removed by scoring with a knife at a distance of at least 0.5 mm from the silicone mold boundary. All films were stored under vacuum in a dry environment before measurement to avoid structural changes.

Films for curcumin loading were directly treated with methanolic curcumin. Films for diazonium coupling and anthocyanin loading were weighed and annealed with 80% v/v methanol/ultrapure water (10 mL per 0.1 g film) for 0.5–1 h. The films were then dried overnight at room temperature and weighed before diazonium coupling or loading.

Dried films loaded with anthocyanin and curcumin were water annealed in a water-filled vacuum desiccator using an 85.7 kPa vacuum (70% humidity) for at least 6 h at room temperature to produce a water-insoluble, plasticized film. The films were removed from the vacuum desiccator, folded into an origami architecture within 10 min, and allowed to dry for at least 2 h. Films were refolded into alternative shapes by repeating the water annealing process.

### Heterogeneous Diazonium Coupling

A cooled solution of 0.2 M aniline (1.25 mL) in acetonitrile and a 1.6 M aqueous solution of *p*-toluenesulfonic acid (0.625 mL) were combined with a cooled aqueous solution of 0.8 M NaNO_2_ (0.625 mL). The mixture was placed in an ice bath for 15 min with continuous stirring. A silk film in 1:1 acetonitrile/0.1 M borate buffer pH 9 (total solution volume 10 mL/0.1 mg) was combined with the stock diazonium salt solution (∼0.98 equiv with respect to tyrosine, assuming 288 tyrosines and according to a H-chain molecular weight of 391 kDa), and the mixture was placed in an ice bath. After combining the silk and diazonium salt, the reaction was allowed to proceed for 1 h. The film was then treated with ultrapure water (30 mL) for 1 h. This step was repeated two further times before drying the film at room temperature in the dark.

### Extraction of Anthocyanin

Red cabbage (East Lothian, Scotland, Billy Logan, Class 1, 00096, DWW) (400 g) was cut into approximately 5 × 5 mm pieces and boiled, with manual stirring, in ultrapure water (850 mL) at 98–105 °C for 0.5 h. Insoluble matter was separated from the anthocyanin solution with a sieve. The solution was concentrated at 80 °C to a final volume of 200 mL. The solution was left to cool to room temperature and then filtered through 12–15 μm qualitative filter paper (VWR, Radnor, PA, USA) and stored at 4 °C for 17 h before use.

### Anthocyanin Loading

Native silk and azosilk films (6 × 4.5 cm and 6 × 8.5 cm) containing 0.1% iron oxide (w/w) were submerged in the anthocyanin solution (100 mL g^–1^) for 17 h under constant movement on a tilt table at 10 osc min^–1^ at 25 °C. The films were then removed and washed in ultrapure water (100 mL) three times for 20 min each on an orbital shaker at 240 rpm. The films were protected from light throughout the loading and washing process. Finally, the films were left to dry in the dark before being imaged on an iPhone SE (Apple, Cupertino, CA, USA) reverse camera at a focal length of 9.7 cm. Loading was repeated in triplicate for the 6 × 4.5 cm film size.

The photographs were standardized using a Datacolor SpyderCheckr 24 (v1.3, Datacolor, NJ, USA) color chart under the same lighting conditions. The calibration photo was imported to Adobe Lightroom Classic (Adobe, San Jose, CA, USA), the angle corrected, chromatic aberration removed, perspective profile corrected using the auto or full setting, a full transformation completed, and the image cropped. The white balance was altered using cell E2, adjusting the exposure, highlights, shadows, whites, and blacks to achieve RGB values of 90% at cell E2 and 4% at cell E4. The image was then edited in SpyderCheckr using the colorimetric mode. The resulting color profile was applied to all images under the same lighting conditions. The edited images were exported as 300 ppi JPG files, and a grid overlay was placed in ImageJ v1.52n (National Institutes of Health, Bethesda, MD, USA). The RGB values were measured for four boxes on the grid (595,952 pixels), and the averages were calculated.

### Curcumin Loading

Native silk and azosilk fibroin films (6 × 4.5 cm and 6 × 8.5 cm) containing 0.1% iron oxide (w/w) were submerged in a 2.5 mg mL^–1^ solution of curcumin in methanol (100 mL g^–1^) for 30 min under constant movement on a tilt table at 10 osc min^–1^. The films were then removed and washed in ultrapure water (100 mL) three times for 20 min each on an orbital shaker at 240 rpm. The films were protected from light throughout the loading and washing process. Finally, the films were left to dry in the dark before being imaged on an iPhone SE or a OnePlus 8 (48MP, f/1.8 ISO320) reverse camera at a focal length of 9.7 cm. Standardization was undertaken as for anthocyanin loading. Loading was repeated in triplicate for the 6 × 4.5 cm film size.

## Supporting Information Available

Additional experimental details: characterization of silk films, characterization of silk origami boats; Results and Discussion: dimensions of mechanically cut silk fibers; native silk and azosilk responses to aqueous environments; first-cycle thermal analysis data for native silk and azosilk films and representative thermograms; anthocyanin extraction workflow; the change in pixel channel intensities in the RGB color space upon loading and unloading native silk and azosilk films with curcumin and anthocyanin; the change in mean pixel channel intensities in the RGB color space, the color changes and the relative color changes of curcumin and anthocyanin-loaded native silk and azosilk films upon exposure to varying pH, surfactants, and heavy metal salts; the colorimetric stability of curcumin and anthocyanin-loaded native silk and azosilk films determined using digital postprocessing and segmentation, and using ColorAssist Lite; origami folding and smart silk origami workflows (PDF)

## Notes

All data supporting this research are openly available from PURE at the University of Strathclyde: https://doi.org/10.15129/47cd5ff3-126e-4e7a-a7a1-6eca449fd490.

## ABBREVIATIONS

RGB: the RGB color space, red-green-blue
Lab: the CIELAB color space, Lightness-*a* axis-*b* axis
PI: 95% prediction interval
SEM: scanning electron microscopy
