# Supplementary material for: Smart Silk Origami as Eco-sensors for Environmental Pollution
Source: ACS Appl Bio Mater. 2022 May 16;5(8):3658–66. doi: 10.1021/acsabm.2c00023 (PMC9382635; doi:10.1021/acsabm.2c00023)
Supplement: Supplementary file 1 — mt2c00023_si_001.pdf [file mt2c00023_si_001.pdf]

Supporting Information

Smart Silk Origami as Eco-Sensors for Environmental Pollution

*Saphia A. L. Matthew,[a, †] Gemma Egan,[a, †] Kimia Witte,[†] Jirada Kaewchuchuen,[#, †] Suttinee Phuagkhaopong,[†] John D. Totten[†,‡] and F. Philipp Seib *[†,§]*

[†] Strathclyde Institute of Pharmacy and Biomedical Sciences, University of Strathclyde, 161 Cathedral Street, Glasgow, G4 0RE, U.K.

[#] Faculty of Nursing, HRH Princess Chulabhorn College of Medical Science, Chulabhorn Royal Academy, Bangkok, Thailand

[§] EPSRC Future Manufacturing Research Hub for Continuous Manufacturing and Advanced Crystallisation (CMAC), University of Strathclyde, Technology and Innovation Centre, 99 George Street, Glasgow G1 1RD, U.K.

[a] These authors contributed equally to this work.

*Tel: +44 (0) 141 548 2510; E-mail: philipp.seib@strath.ac.uk.

Table of Contents

Experimental Methods **S3**

Characterization of silk films S3

Color change of silk films across pH and pollutant concentration S3

Colorimetric stability of native silk and azosilk films S4

Scanning electron microscopy S4

Contact angle measurement S5

Fourier Transform Infrared Spectroscopy analysis S5

Thermal analysis S6

Swelling analysis S6

Electromagnetic field strength S6

Contraction and relaxation on water S7

Thickness and folding assessment S7

Characterization of silk origami boats S7

Semi-autonomy S7

Sensing of pH and pollution S8

Statistical analysis and graphic design S8

Results and Discussion **S10**

Figure S1 .... S10

Figure S2 .... S11

Table S1 .... S12

Table S2 .... S13

Figure S3 .... S14

Figure S4 .... S16

Figure S5 .... S17

Figure S6 .... S18

Figure S7 .... S19

Figure S8 .... S20

Figure S9 .... S21

Figure S10 .... S23

Figure S11 .... S25

Figure S12 .... S26

Figure S13 .... S27

References......S27

Experimental Methods

Characterization of silk films

Color change of silk films across pH and pollutant concentration

Silk films loaded with curcumin or anthocyanin were imaged after being submerged for 2 min in the following solutions: 0.2 M potassium phosphate buffer (pH 4.37, pH 6.16, pH 7.00, pH 8.16 and pH 9.15); 0.001 M potassium phosphate buffer (pH 1.98 and pH 2.92); 0.17 M sodium acetate buffer (pH 5.01); 0.1 M sodium bicarbonate buffer (pH 9.97); 0.2 M alkaline borate buffer (pH 10.50); 0.1 M glycine buffer (pH 11.36); 897 mg L^{-1} SDS; 660 mg L^{-1} CTAB; 370 mg L^{-1} Triton X-100; 242.3 mg L^{-1} $CoCl_2$; 341.5 mg L^{-1} $Co(NO_3)_2$; 3.265 mg L^{-1} $CuSO_4$; 0.15748 mg L^{-1} $AgNO_3$; 979.2 mg L^{-1} $MgCl_2$; 999.2 mg L^{-1} $MgCO_3$; 600.0 mg L^{-1} $MgOH_2$; or real, stagnant river water randomly grab sampled in Glasgow, Scotland. The images were processed as for loading. Films were washed three times in ultrapure water for 0.25 h, dabbed dry with a tissue and dried at room temperature for 0.17 h before submersion in a new solution. The relative color change (S) was calculated by subtracting the mean intensities in the red (R_o), green (G_o), and blue (B_o) channels of the loaded film from the red (R), green (G) and blue (B) intensities in the films following exposure to each medium, according to equation 1.[1]

$$S\ (\%) = 100\ \times\ \frac{(|R-R_o|+|G-G_o|+|B-B_o|)}{R+G+B}\ (1)$$

The color change was also calculated based on the Lab model, according to equation 2.[1]

$$\Delta E = \sqrt{(L - L_o)^2 + (a - a_o)^2 + (b - b_o)^2} \quad (2)$$

In the Lab color space published by the International Commission on Illumination (CIE), the L*, a*, and b* channels constitute image lightness, red to green intensities, and blue intensity, respectively. Each condition was repeated with three films.

Colorimetric stability of native silk and azosilk films

Native silk and azosilk films doped with 0.1% (w/w) iron oxide and loaded with curcumin or anthocyanin were stored for 31 days in vacuum sealed boxes at 4 °C or 20 °C in the dark. Silk films were then imaged before and after being submerged in 0.2 M potassium phosphate buffer (pH 9.15) for 2 min on an iPhone SE (Apple, Cupertino, CA, U.S.A.) reverse camera at a focal length of 9.7 cm. Photographs were standardised as described for loading, the RGB values measured for 2 boxes on the grid (42840 pixels) and the averages were calculated. Each condition was repeated with three films.

Digital image colorimetry was also achieved using the ColorAssist Lite© (FTLapps, Inc., Broadlands, VA, U.S.A.) smartphone application with a spatial sample aperture of 50 × 50 pixels, temporal sample aperture of 30 frames (1 second) and mean output intensities measured in the RGB color space. Each film was sampled in three regions and the average calculated. Each condition was repeated with three films.

Scanning electron microscopy

For surface imaging, samples were added to aluminium stubs with sticky carbon tabs, with the surface uppermost. For section imaging, a titling SEM stub was used to rotate the samples to 90° with the cross-section uppermost. Samples were sputter coated (ACE200, Leica Microsystems,

Wetzlar, Germany) with a 20 nm gold layer to minimise charging in the SEM. Samples were viewed with a TM4000Plus SEM (Hitachi Ltd., Tokyo, Japan) operated at beam voltage 10000eV, probe current setting 2, standard vacuum level (M) and with data collected in backscattered electron mode at magnifications of 100×, 1000× and 10000×.

Contact angle measurement

The films were placed on a glass slide. The contact angle was measured using a DSA30 drop shape analyser (KrussGmbH, Hamburg, Germany) equipped with a manual syringe and needle (diameter 0.8 mm). Droplet size was controlled manually. Results were analysed in Advance software (KrussGmbH, Hamburg, Germany) with manual droplet shape fitting.

Fourier Transform Infrared Spectroscopy analysis

Positive silk II controls were provided by autoclaved silk films and silk films treated with 70% v/v ethanol/ultrapure H_2O, while air-dried silk films and freeze-dried silk were used as positive controls for silk I structure. Secondary structures of silk films, freeze-dried powders and freeze-dried particles were analysed by Fourier transform infrared spectroscopy (FTIR) on an ATR-equipped TENSOR II FTIR spectrometer (Bruker Optik GmbH, Ettlingen, Germany). Each FTIR measurement was recorded in absorption mode over the wavenumber range of 400 to 4000 cm^{-1} at 4 cm^{-1} resolution for 128 scans and then corrected for atmospheric absorption using Opus (Bruker Optik GmbH, Ettlingen, Germany). The second derivative of the background-corrected FTIR absorption spectra was analysed in OriginLab 19b® (Northampton, MA, U.S.A.) by adapting a literature protocol.[2] The second derivative was smoothed twice using a seven-point Savitzky-Golay function with a polynomial order of 2. The amide I region was analysed by interpolation of a non-zero linear baseline between 2–3 of the highest values in the 1600–1700 cm-

^{1} range. Peak positions were identified by applying the second derivative, and the peaks were fitted in the amide I region using non-linear least squares with a series of Gaussian curves. The position, width and height of each peak were allowed to vary, while peak area could take any value less than or equal to 0. Deconvoluted spectra were then area-normalised, and the relative area of each band was used to calculate the secondary structure content according to literature band assignments.[3,4]

Thermal analysis

First-cycle differential scanning calorimetry and thermogravimetric analysis were carried out on the dried samples (3–5 mg) in aluminium pans from 20–350 °C at a scanning rate of 10 °C min^{-1} and under a nitrogen flow of 50 mL min^{-1} using an STA Jupiter 449 (Netzsch, Gerätebau GmbH, Selb, Germany). Thermograms were analysed using Proteus® (Netzsch, Gerätebau GmbH, Selb, Germany).

Swelling analysis

The swelling of silk fibroin films was monitored over 30 min. Each film was split into 3 pieces and placed in ultrapure water (20 mL), and the weight was measured at defined intervals. The films were removed, and any excess water on the film was dabbed dry with paper towels. This was repeated three times for each film.

Electromagnetic field strength

The strength of a magnetic field was measured by the ability to pull a floating rectangular silk film (0.1 g, 25 μm thickness) along the water-air interphase using a N52 round cylinder magnet (25 × 20 mm Rare Earth Neodymium; $(BH)_{max}$ 52 MGOe). The magnet used in the study was moved across water-glass interphase (2 mm thickness) parallel to the water-air interphase (where silk was

floating) at fixed distances defined by the volume of water in a 5000 mL glass beaker. Every added 50 mL was equivalent to a 1 mm increase in the distance between the silk film and the magnet. The distance from the surface of the magnet at which locomotion was first observed was converted to magnetic flux density (B) and magnetic field strength (H).

Contraction and relaxation on water

The time of contraction from the original shape of the water-annealed dried silk films (*ca*. 7 × 5 cm) was measured in seconds as the films were placed in water (floating at the water-air interface). The relaxation and return to the original shape were followed by measuring time in seconds.

Thickness and folding assessment

Film thickness was measured with digital Vernier callipers (CM145 Clarke® Precision, Clarke®, London, U.K.) by stacking 3–4 films of the same type. The folding time after annealing for 17 h was measured by removing the films (ca. 9 × 13.5 cm) from the water annealing chamber and recording the time when the films first cracked or tore when being repeatedly folded and unfolded by hand.

Characterization of silk origami boat

Semi-autonomy

Origami sailboats (6 × 8.5 cm) containing 0.1% (w/w) iron oxide were placed on ultrapure water in a clear acrylic box (10.3 × 10.3 × 5 cm). An LED panel (RALENO, Seattle, WA, U.S.A.) was fixed at the back of the box to provide constant illumination at 5600K color temperature and at 1% brightness. An N52 round cylinder magnet (25 × 20 mm Rare Earth Neodymium) was suspended

from the bow of the boat at ca. 2 mm horizontally and from the highest point of the boat sail at ca. 2 mm vertically. The distance travelled over 30 s was captured on an iPhone SE (Apple, Cupertino, CA, U.S.A.) reverse camera at a capture speed and resolution of 240 fps and 1080 p using FiLMiC Pro (FiLMiC Inc., Seattle, WA, U.S.A.). In parallel, the movement of a folded origami boat manufactured without iron oxide was used as a negative control. Each condition was repeated with three origami boats manufactured with different silk batches. Images were extracted at 240 fps using FFmpeg.[5]

Sensing of pH and pollution

Films (4.5 × 4.5 cm) were loaded with anthocyanin and curcumin, water annealed and folded into origami canoes. Boat hulls were imaged on an iPhone SE (Apple, Cupertino, CA, U.S.A.) reverse camera at a focal length of 7.8 cm. Curcumin-silk canoes were placed on 0.2 M potassium phosphate buffer (pH 4.73 and 9.15), and anthocyanin-silk canoes were placed on 660 mg L^{-1} CTAB and 0.2 M potassium phosphate buffer (pH 9.15) for 2.5 mins before removal, drying in air and imaging. Photographs were standardised as described for loading of 2D films, the RGB values were measured for 2 boxes on the grid (42840 pixels) and the averages were calculated.

Equivalent origami sailboats (6 × 8.5 cm) were placed on 0.2 M potassium phosphate buffer (pH 9.15), 897 mg L^{-1} SDS and 3.27 mg L^{-1} copper sulphate in a clear acrylic box (10.3 × 10.3 × 5 cm). An LED panel (RALENO, Seattle, WA, U.S.A.) was fixed at the back of the box to provide constant illumination at 5600K color temperature and at 1% brightness. The color change was imaged on an iPhone SE (Apple, Cupertino, CA, U.S.A.) reverse camera at a capture speed and resolution of 60 fps and 2160 p using FiLMiC Pro (FiLMiC Inc., Seattle, WA, U.S.A.). Images were extracted at 60 fps using FFmpeg.[5]

Statistical analysis and graphic design

Data were analysed using Microsoft Excel® 2019 (Microsoft Office 365 ProPlus Software, Redmond, WA, U.S.A), Minitab® (Minitab® Statistical Software, State College, PA, U.S.A.) and GraphPad Prism 8.2.1 (GraphPad Software, La Jolla, CA, U.S.A.). Normality of the data distributions and equivalence of variance for sample pairs and multiple groups were assumed throughout. Two sample groups were analysed using the independent *t*-test. A two-way ANOVA was used to compare multiple groups across two independent variables, followed by Šidák's multiple comparison, simple effects post-hoc test. Stability was analyzed using a two-way ANOVA to compare multiple groups across the storage temperature and modification as independent variables, followed by Dunnett's multiple comparison, simple effects post-hoc test against the t = 0-day control. Cubic polynomials were fitted to the color change (ΔE) data between pH values of 1.98 to 11.36 using non-linear, least squares regression in GraphPad Prism 8.2.1 (GraphPad Software, La Jolla, CA, U.S.A.) and the 95% prediction interval of each equation was calculated. Curve fitting used no special handling for outliers, medium convergence criteria, no weighting, and considered each replicate ΔE value as an individual point. Statistical significance, identified using post-hoc tests, was as follows: *p < 0.05, **p < 0.01, ***p < 0.001, ****p < 0.0001. All data are displayed as the mean ± standard deviation, with the number of experimental repeats (*n*) shown in each figure legend. Illustrations were made in Adobe Illustrator (1.8.2) (Adobe, San Jose, CA, U.S.A.) and ChemBioDraw® 20.0 (PerkinElmer, Waltham, MA, U.S.A.) software.

Results and Discussion

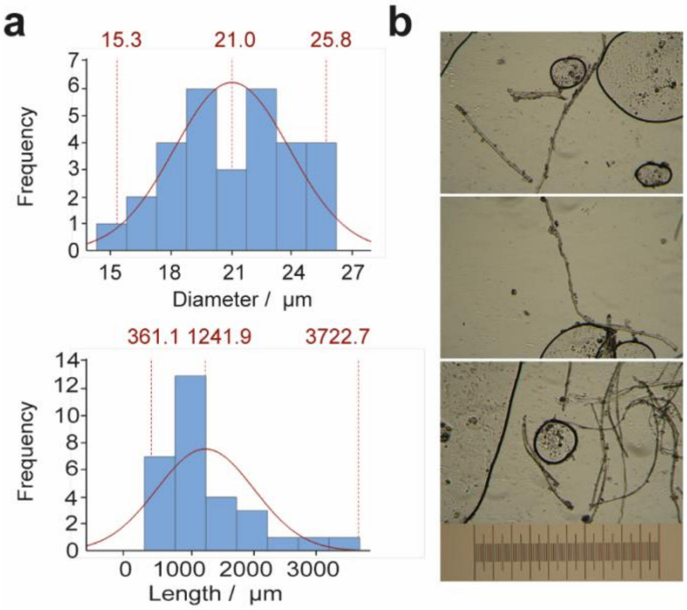

Figure S1. The dimensions of mechanically cut silk fibers. **(a)** Histograms of length and width. Reference lines show the minimum, average and maximum dimensions. $n = 30$. **(b)** Exemplar images of silk fibers at 10× magnification. Scale bar = 1 mm.

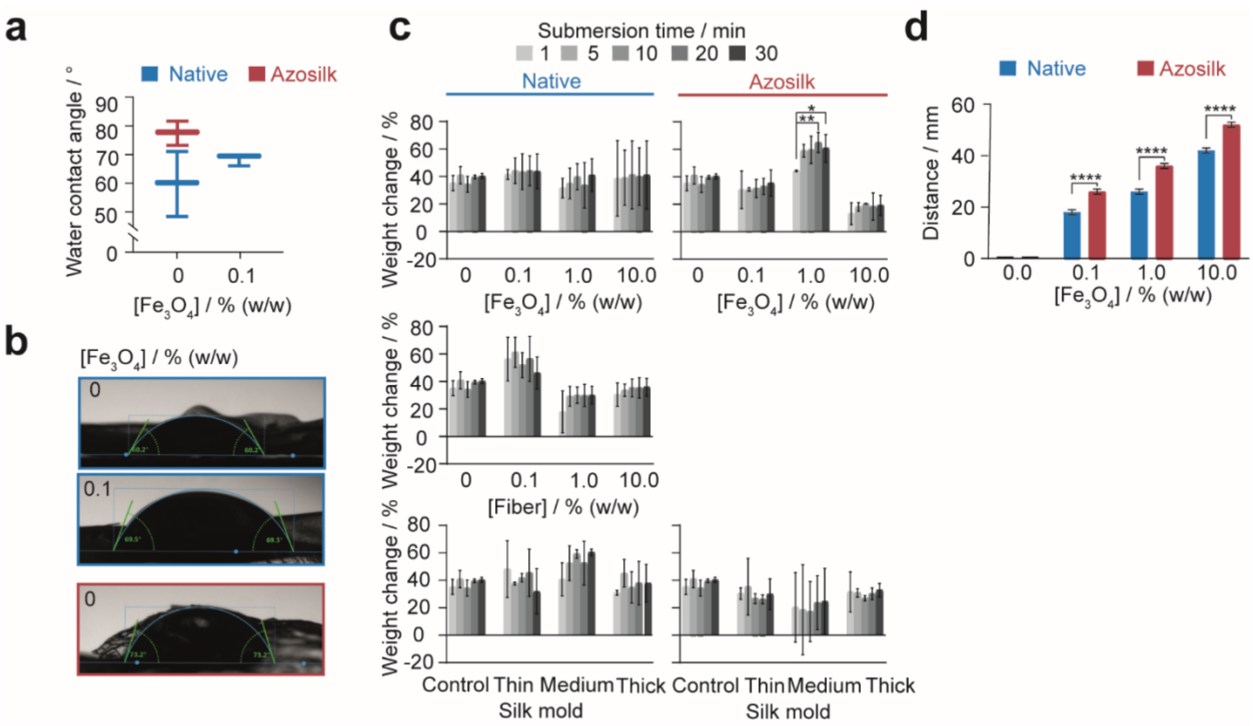

Figure S2. The effect of aqueous environments on silk films. **(a)** Water contact angle of azosilk and native silk medium thickness films without iron oxide and of native control thickness silk film containing 0.1 % (w/w) iron oxide. ± SD, $n = 3$. **(b)** Exemplar images of water contact angles for azosilk and native silk films. Scale bars = 0.5 cm. **(c)** Weight change of native silk and azosilk films following immersion in ultrapure water. **(d)** The distance from the surface of an N52 Rare Earth Neodymium round cylinder magnet that was able to pull a floating rectangular-shaped silk film (0.1 g, 25 µm) loaded with iron oxide particles along the water-air interface. ± SD, $n = 3$. Error bars are hidden in the bars and plot symbols when not visible. Multiple factors were evaluated by two-way analysis of variance (ANOVA), followed by Šidák's multiple comparison, simple effects post-hoc test. Asterisks denote statistical significance determined using the *t*-test and post-hoc tests as follows: *$p < 0.05$, **$p < 0.01$, ***$p < 0.001$, ****$p < 0.0001$.

Table S1. First-cycle thermal analysis data of native silk films with different thickness, fiber contents and iron oxide particle contents. ± SD, *n = 3*.

	Thermal Property	**Thickness** / *μm*			**[silk fiber]** / *% (w/w)*			**[Fe_3O_4]** / *% (w/w)*			**-Silk II**
		Thin	Medium	Thick	0.1	1	10	0.1	1	10	Silk film
**DSC**	T_g / °C	53.6 ± 0.8[a]	53.9 ± 17.5	63.3 ± 27.2	50.9 ± 18.5	47.5 ± 9.6	52.3 ± 5.7	50.4 ± 5.5	61.9 ± 25.3	50.2 ± 5.4	50.3 ± 4.9
	T_d / °C	61.2 ± 3.1	66.7 ± 7.9	61.3 ± 7.0	78.2 ± 22.0	70.2 ± 20.5	61.3 ± 6.4	66.0 ± 17.7	61.4 ± 6.1	65.6 ± 17.5	60.4 ± 8.6
	ΔH_d / $J\ g^{-1}$	-221. 5 ± 19.5	-184.8 ± 41.6	-203.4 ± 40.6	-37.9 ± 17.5	-122.8 ± 54.2	-84.8 ± 52.9	-98.8 ± 20.7	-177.5 ± 15.3	-104.4 ± 27.6	-123.0 ± 103.4
	T_g' / °C	200.6 ± 4.5	200.2 ± 4.8	202.9 ± 3.3	194.7[b]	202.0[b]	204.8 ± 3.3[a]	203.4 ± 3.7	202.8 ± 1.7	198.6 ± 4.0	-
	T_c / °C	-	235.1 ± 3.0[b]	237.5 ± 2.4	228.4± 5.1[a]	-	237.0 ± 9.4[a]	236.3 ± 0.0[a]	235.8 ± 1.3[a]	229.6[b]	233.6[b]
	ΔH_c / $J\ g^{-1}$	-	3.18 ± 3.63[a]	5.25 ± 3.72	1.61 ± 0.30[a]	-	0.885 ± 0.447[a]	5.92 ± 0.80[a]	1.61 ± 0.82[a]	4.32[b]	3.20[b]
	T_o / °C	258.3 ± 0.3	258.4± 0.8	258.7 ± 0.5	253.4 ± 3.4	254.3 ± 0.7	253.1 ± 2.1	256.5 ± 0.9	260.9 ± 3.6	253.9 ± 1.6	257.0 ± 1.9
	T_{dec} °C	277.0 ± 0.06	277.5 ± 0.0[a]	278.9 ± 0.30	277.0 ± 0.4	276.1 ± 0.36	275.9 ± 2.1	277.1 ± 0.6	278.5[b]	277.5 ± 0.7	277.8 ± 1.0
	T_{dec}' / °C	270.5 ± 1.7	271.8 ± 1.7	272.1 ± 1.1	267.2 ± 3.1	268.3 ± 2.0	264.2 ± 5.1	270.9 ± 1.1	274.9 ± 3.4	269.8 ± 5.8	269.6 ± 2.2
**TGA**	**Water content** / *% (w/w)*	6.5 ± 0.5	6.6 ± 1.6	6.1 ± 1.9	5.6 ± 0.4	5.9 ± 0.1	5.4 ± 1.6	4.9 ± 0.5	5.4 ± 0.4	3.9 ± 0.7	3.7 ± 0.6
	T_o / °C	263.6 ± 1.1	263.9 ± 0.4	262.7 ± 0.7	264.0 ± 1.6	264.8 ± 1.3	262.4 ± 1.2	264.0 ± 0.5	263.8 ± 1.5	264.4 ± 0.8	262.8 ± 1.7
	T_{dec}' / °C	278.3 ± 0.2	278.3 ± 1.0	279.0 ± 0.6	278.5 ± 0.6	278.8 ± 0.4	275.9 ± 2.7	278.8 ± 0.5	279.1 ± 0.3	278.8 ± 0.1	278.6 ± 0.6

a. *n = 2*

b. *n = 1.*

Table S2. First-cycle thermal analysis data of azosilk films with different thickness, fiber contents and iron oxide particle contents. ± SD, *n = 3.*

	Thermal Property	**Thickness** / *µm*			**[silk fiber]** / *% (w/w)*			**[Fe_3O_4]** / *% (w/w)*			**-Silk II**
		Thin	Medium	Thick	0.1	1	10	0.1	1	10	Silk film
**DSC**	**T_g** / *°C*	65.3 ± 4.0[a]	50.1 ± 0.1	75.6 ± 10.8	76.3 ± 12.3[a]	73.1 ± 6.4	71.5 ± 9.1	60.7 ± 23.5	54.7 ± 13.0	67.5 ± 18.9	38.7 ± 6.1[a]
	**T_d** / *°C*	81.9 ± 5.5	56.8 ± 0.7	71.1 ± 6.2	74.3 ± 1.8	71.6 ± 12.7	67.0 ± 2.0	62.1 ± 9.2	62.6 ± 8.6	66.0 ± 7.3	67.5 ± 4.1
	**ΔH_d** / *$J\ g^{-1}$*	-126.0 ± 90.3	-138.9 ± 41.6	-93.6 ± 53.6	-115.9 ± 53.7	-166.3 ± 31.7	-134.7 ± 29.8	-122.4 ± 12.5	-167.2 ± 80	-124.1 ± 62.1	-156.6 ± 39.5
	**T_g’** / *°C*	200.1 ± 1.1[a]	201.1[b]	201.0 ± 0.1[a]	200.0 ± 1.3[a]	197.6 ± 1.9	200.6 ± 5.2[a]	200.6 ± 0.4[a]	200.1[b]	200.2 ± 0.7	202.1 ± 0.5
	**T_c** / *°C*	237.9 ± 0.7[b]	253.7 ±2.4[b]	235.9 ± 2.2[b]	237.0[b]	228.6[b]	239.1	-	-	-	-
	**ΔH_c** / *$J\ g^{-1}$*	1.02 ± 1.17[b]	1.06 ± 0.23[b]	0.333 ± 0.472[b]	0.479[b]	0.185[b]	0.336[b]	-	-	-	-
	**T_o** / *°C*	264.4 ± 2.5	258.8 ± 2.9	262.3 ± 4.5	262.3 ± 1.0	265.2 ± 1.0	265.1 ± 3.6	262.5 ± 1.7	264.9 ± 1.6	264.7 ± 2.0 0.30	265.4 ± 1.5
	**T_{dec}** *°C*	279.3[b]	279.0 ± 0.6[a]	-	-	-	-	-	-	-	-
	**T_{dec}’** / *°C*	272.9 ± 3.2	270.4 ± 1.4	274.5 ± 3.3	272.9 ± 3.3	277.3 ± 1.9	277.0 ± 5.0	271.9 ± 4.4	276.0 ± 4.5	277.4 ± 2.8	278.8 ± 0.1
**TGA**	**Water content** / *% (w/w)*	5.7 ± 0.6	4.0 ± 0.7	6.0 ± 1.1	5.8 ± 0.3	7.2 ± 1.0	7.7 ± 0.8	5.0 ± 1.6	4.8 ± 1.4	4.4 ± 0.8	7.2 ± 1.6
	**T_o** / *°C*	262.9 ± 0.5	261.6 ± 0.3	261.4 ± 1.4	263.3 ± 0.6	262.5 ± 1.7	262.2 ± 0.5	263.5 ± 0.3	263.4 ± 1.0	263.5 ± 2.6	263.8 ± 1.8
	**T_{dec}’** / *°C*	279.1 ± 0.2	269.4 ± 11.2	276.1 ± 4.5	279.1 ± 0.2	278.5 ± 0.7	277.6 ± 1.9	279.0 ± 0.2	279.0 ± 0.2	279.0 ± 0.4	278.4 ± 0.4

a. *n = 2*

b. *n = 1.*

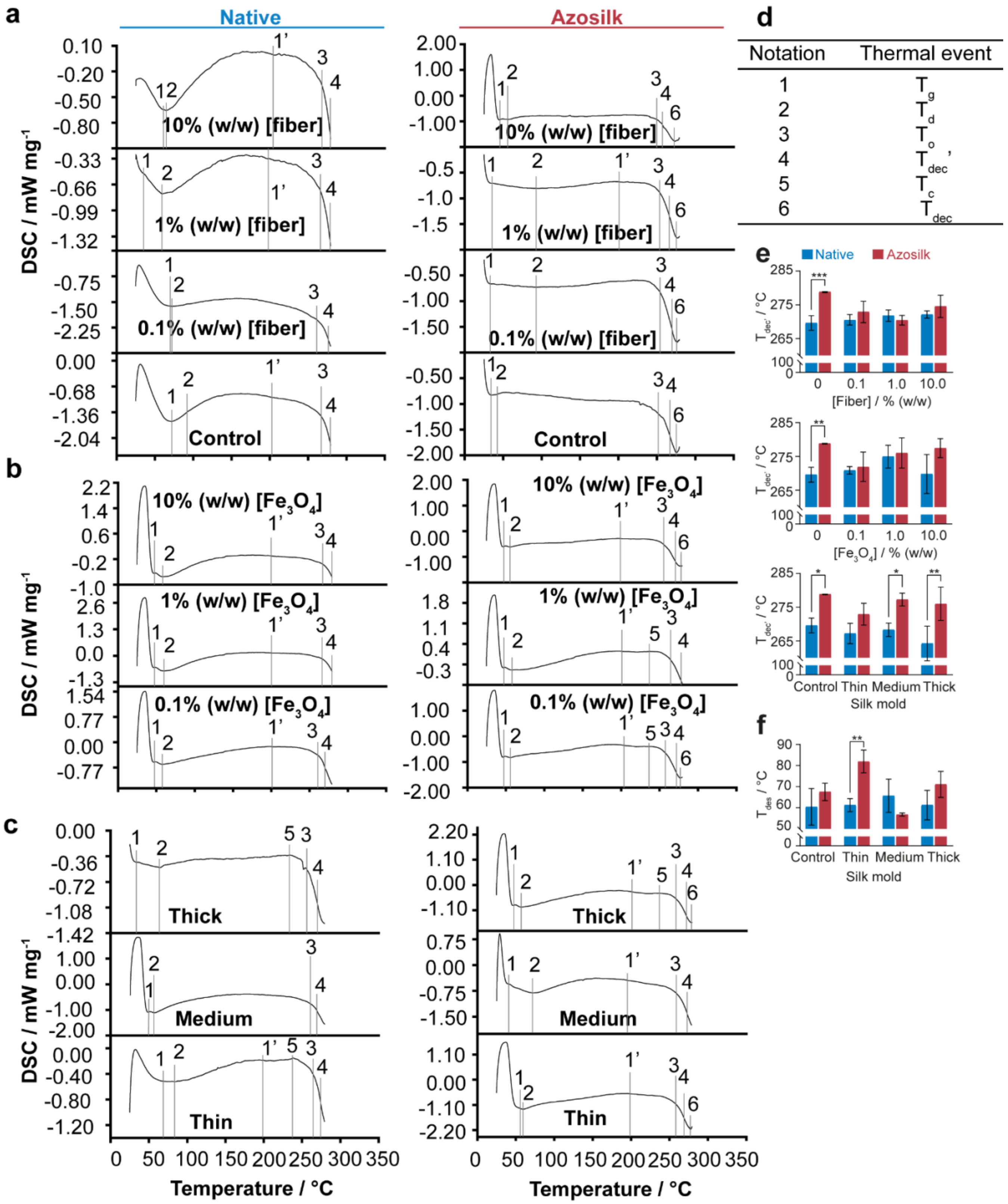
a
Native
Azosilk
DSC / mW mg-1
10% (w/w) [fiber]
1% (w/w) [fiber]
0.1% (w/w) [fiber]
Control
b
10% (w/w) [Fe3O4]
1% (w/w) [Fe3O4]
0.1% (w/w) [Fe3O4]
c
Thick
Medium
Thin
Temperature / °C
d
Notation Thermal event
1 Tg
2 Td
3 To
4 Tdec'
5 Tc
6 Tdec
e
Native
Azosilk
Tdec' / °C
[Fiber] / % (w/w)
[Fe3O4] / % (w/w)
Control Thin Medium Thick
Silk mold
f
Tdes / °C
Control Thin Medium Thick
Silk mold

Figure S3. Representative first-cycle raw differential scanning calorimetry (DSC) of silk films manufactured with **(a)** different silk fiber concentrations, **(b)** iron oxide particle concentrations and **(c)** thicknesses. **(d)** Thermal event assignment key. **(e)** The temperature of maximum decomposition rate and **(f)** the water desorption temperature of silk films. ± SD, n = 3. Water desorption temperature ($T_{d(es)}$), glass transition temperatures (T_g), extrapolated onset temperature of decomposition (T_o), crystallization temperature (T_c), decomposition temperature (T_{dec}) and the temperature of maximum decomposition rate (T_{dec}') are reported. Error bars are hidden in the bars and plot symbols when not visible. Multiple factors were evaluated by two-way analysis of variance (ANOVA), followed by Šidák's multiple comparison, simple effects post-hoc test. Asterisks denote statistical significance determined using the post-hoc tests as follows: *p < 0.05, **p < 0.01, ***p < 0.001, ****p < 0.0001.

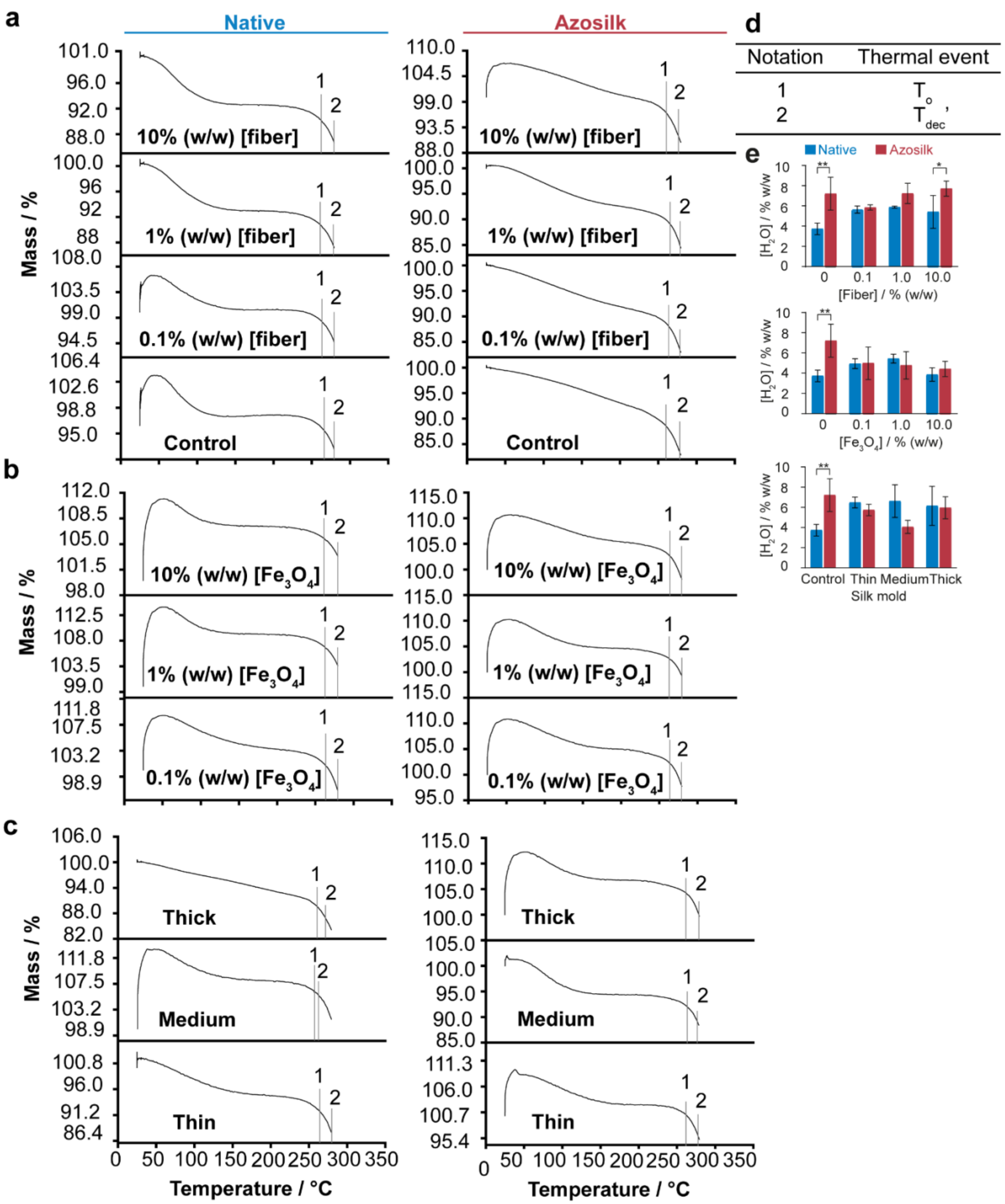

Figure S4. Representative first-cycle thermogravimetric analysis (TGA) thermograms of silk films manufactured with **(a)** different silk fiber concentrations, **(b)** iron oxide particle

concentrations and **(c)** thicknesses. **(d)** Thermal event assignment key. **(e)** Composition of water (% (w/w)) in silk films determined as the percentage mass loss at 140 °C. ± SD, *n* = 3. Extrapolated onset temperature of decomposition (T_o) and the temperature of maximum decomposition rate (T_{dec}') are reported. Error bars are hidden in the bars and plot symbols when not visible. Multiple factors were evaluated by two-way analysis of variance (ANOVA), followed by Šidák's multiple comparison, simple effects post-hoc test. Asterisks denote statistical significance determined using the post-hoc tests as follows: *p < 0.05, **p < 0.01, ***p < 0.001, ****p < 0.0001.

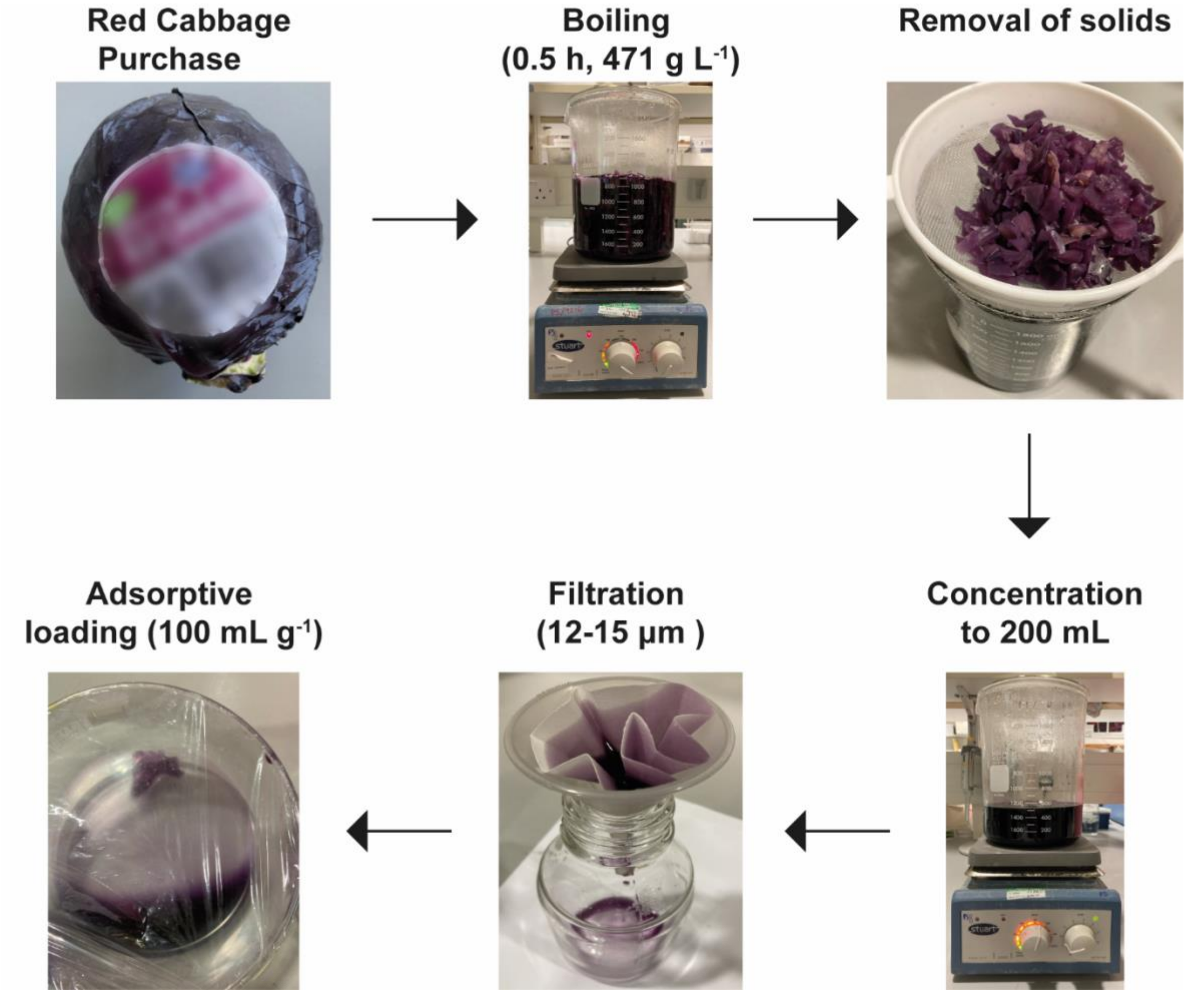

Figure S5. Anthocyanin extraction and silk film loading from fresh red cabbage.

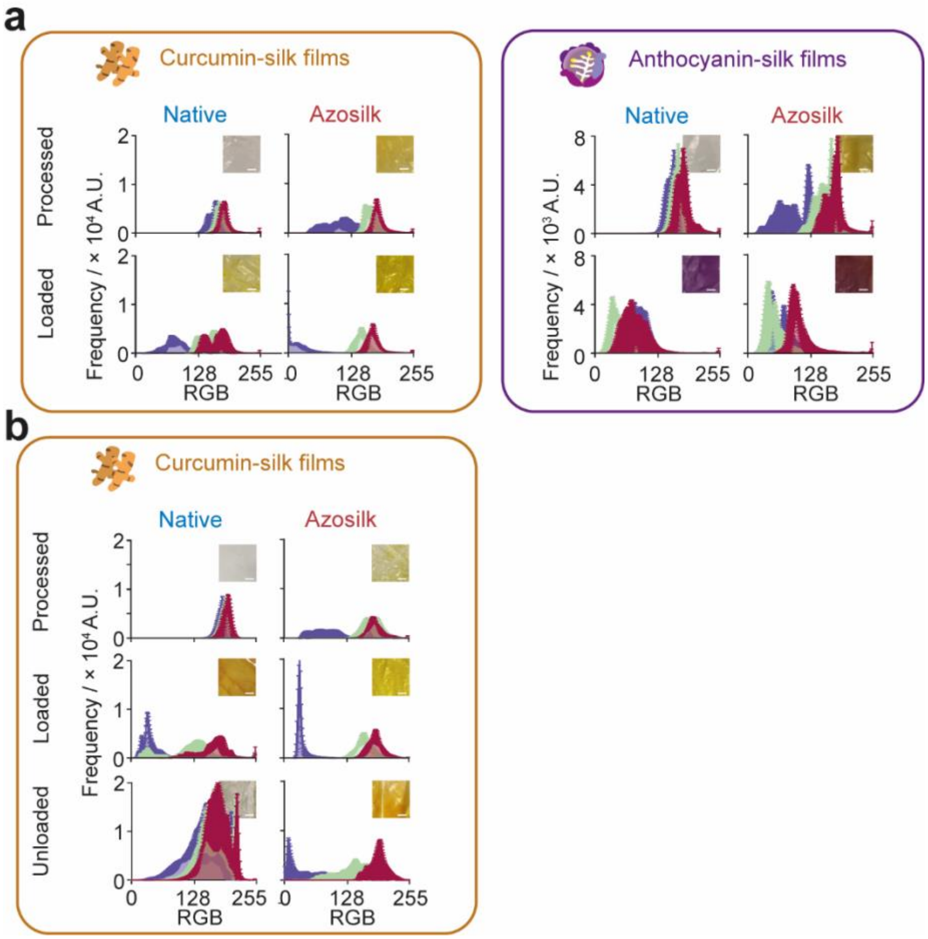

Figure S6. The change in pixel channel intensities upon **(a)** loading medium thickness films containing 0.1% (w/w) iron oxide particles with anthocyanin for sensing pH, heavy metal salts and surfactants and with curcumin for sensing heavy metal salts and surfactants. **(b)** Medium thickness films containing 0.1% (w/w) iron oxide particles were loaded with curcumin for sensing pH and unloaded in 0.1 M aqueous NaOH. Scale bars = 0.5 cm ± SD, *n* = 3.

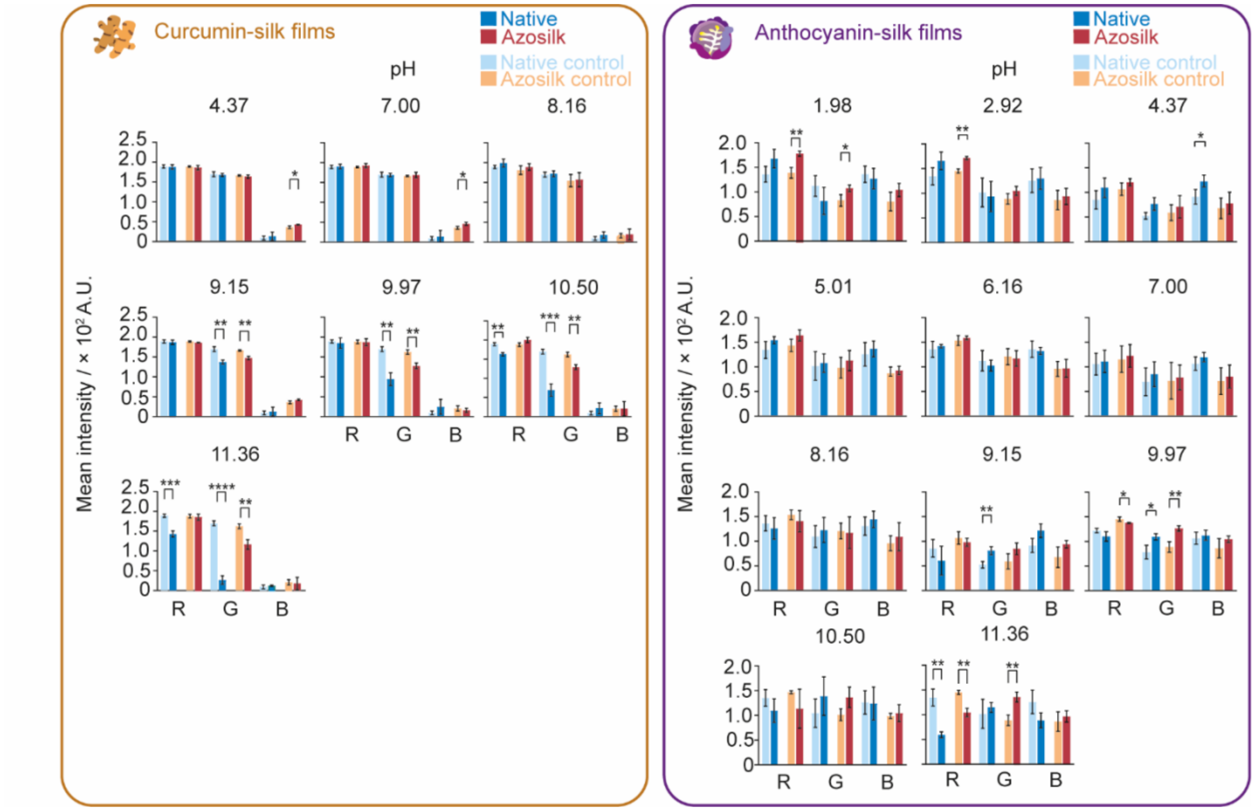

Figure S7. The change in mean pixel channel intensities in the RGB color space of curcumin-loaded and anthocyanin-loaded azosilk and native silk medium thickness films containing 0.1% (w/w) iron oxide particles in response to pH. ± SD, n = 3. Sample pairs were analyzed using the independent t-test. Asterisks denote statistical significance determined using the t-test as follows: *$p < 0.05$, **$p < 0.01$, ***$p < 0.001$, ****$p < 0.0001$.

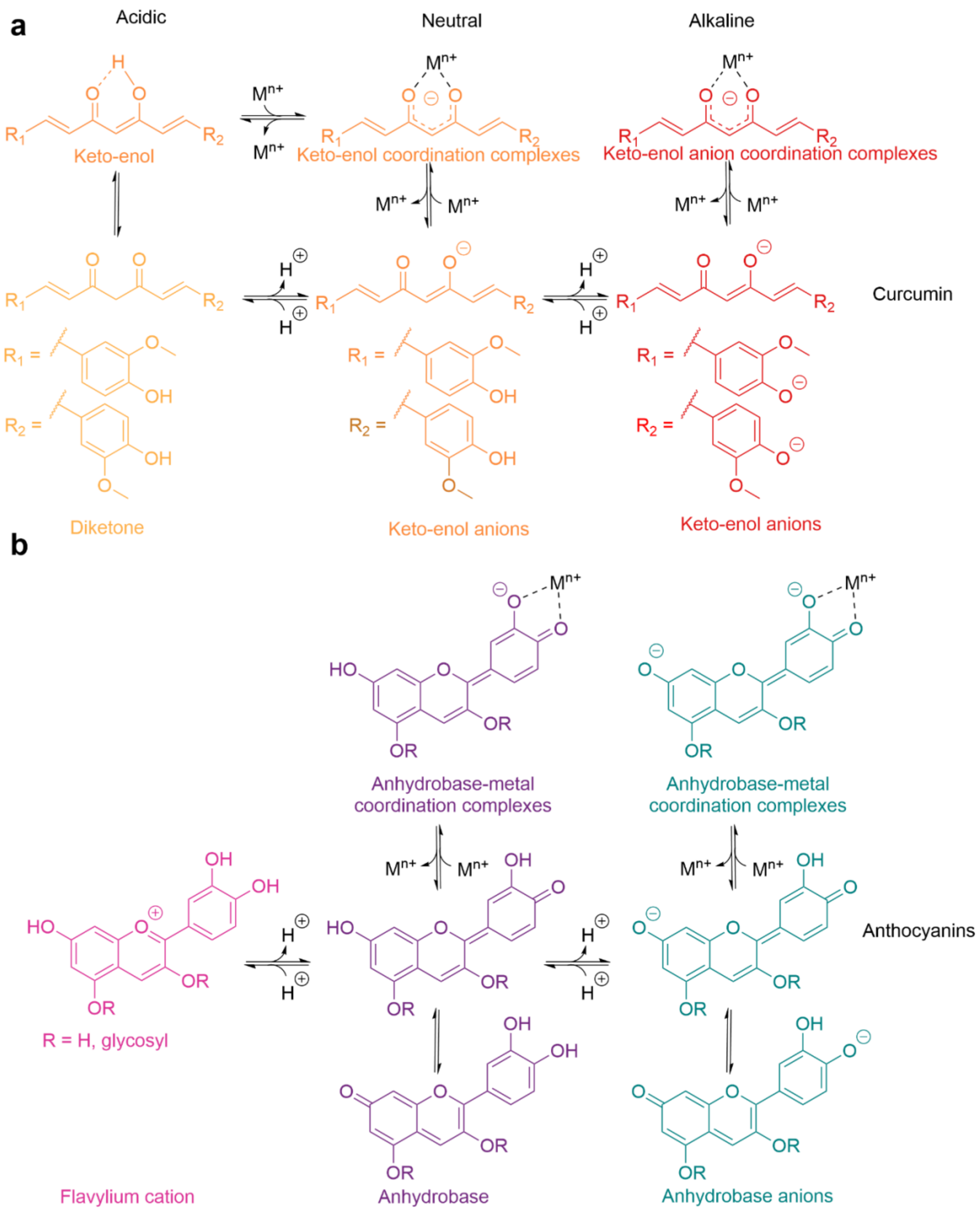

Figure S8. The reversible transformations and color changes of **(a)** curcumin[6,7] and **(b)** anthocyanins[6,8,9] as pH of the surrounding media is raised, from acidic to neutral and to alkaline conditions.

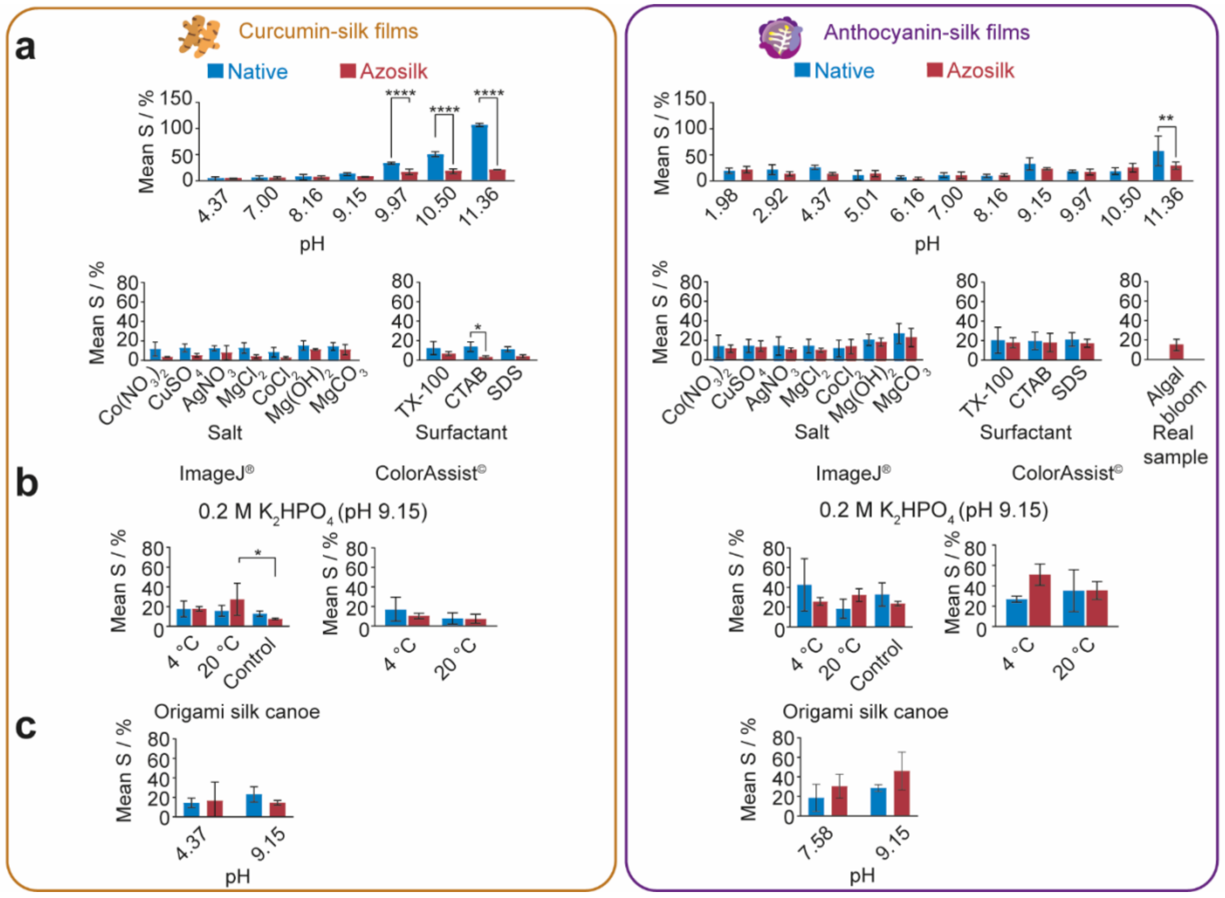

Figure S9. The relative color changes of **(a)** curcumin and anthocyanin-loaded native silk and azosilk films varying pH, surfactant, and heavy metal salt. **(b)** The storage stability of native silk and azosilk film relative color changes following 31 days of storage under vacuum at 4 or 20 °C using image pre-processing and ImageJ® or ColorAssist Lite© to measure mean RGB intensities. **(c)** The relative color changes of curcumin and anthocyanin-loaded native silk azosilk origami canoes varying pH. ± SD, n = 3. Multiple factors were evaluated by two-way analysis of variance (ANOVA), followed by Šidák's multiple comparison, simple effects post-hoc test. Stability was evaluated against the t = 0-day control using a two-way ANOVA followed by Dunnett's multiple comparison, simple effects post-hoc test. Asterisks denote statistical

significance determined using the *t*-test and post-hoc tests as follows: *p < 0.05, **p < 0.01, ***p < 0.001, ****p < 0.0001.

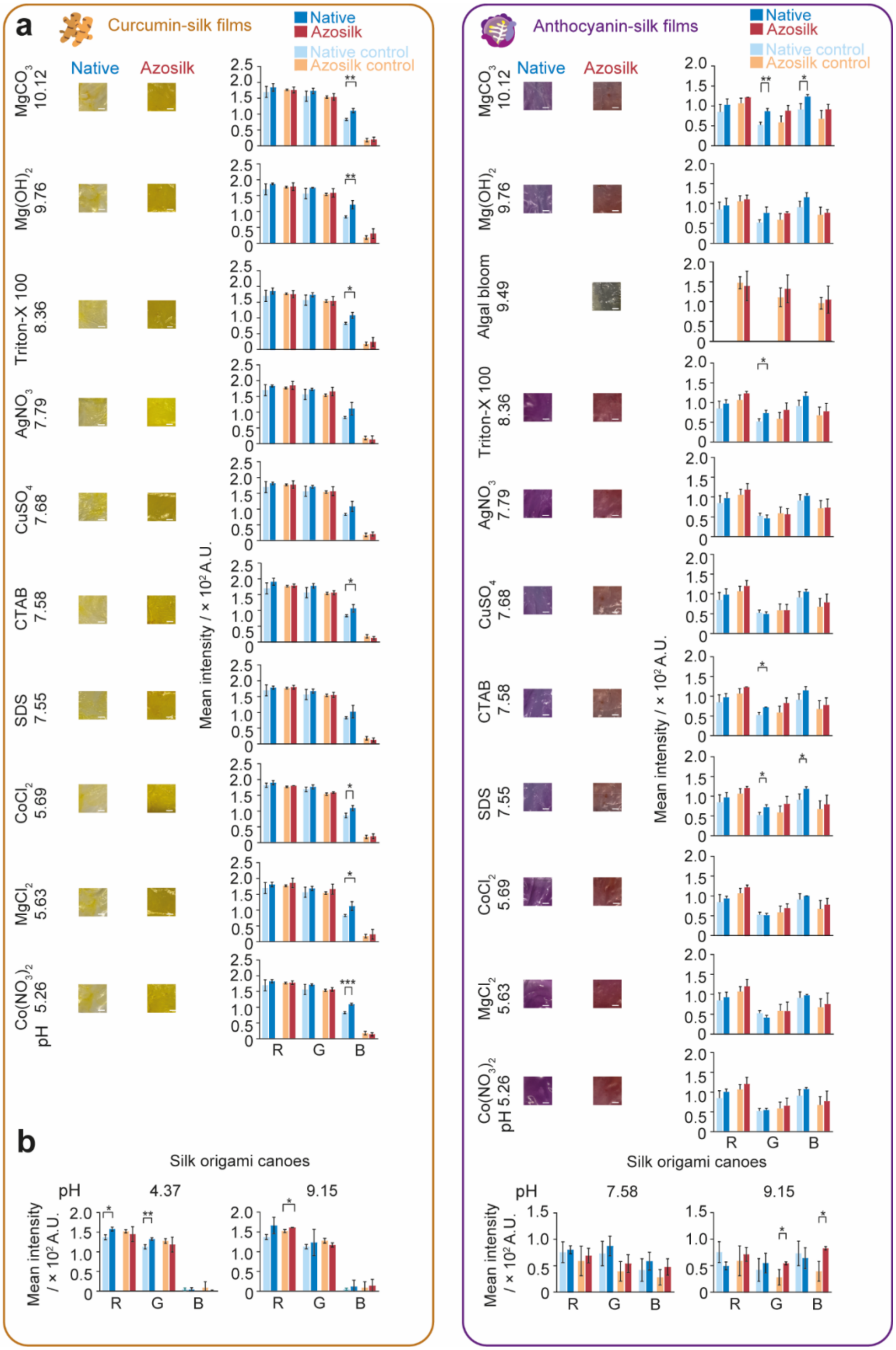
a
Curcumin-silk films
Native
Azosilk
Native control
Azosilk control
Native
Azosilk
MgCO3 10.12
Mg(OH)2 9.76
Triton-X 100 8.36
AgNO3 7.79
CuSO4 7.68
CTAB 7.58
SDS 7.55
CoCl2 5.69
MgCl2 5.63
Co(NO3)2 pH 5.26
Mean intensity / × 10² A.U.
R
G
B
Anthocyanin-silk films
Algal bloom 9.49
b
Silk origami canoes
pH
4.37
9.15
7.58
Mean intensity / × 10² A.U.

Figure S10. The change in color and mean pixel channel intensities of curcumin-loaded and anthocyanin-loaded azosilk and native silk medium thickness films containing 0.1% (w/w) iron oxide particles after exposure to **(a)** surfactants and heavy metal salts serving as model pollutants, and **(b)** after folding into 3D origami silk canoes and exposure to media at the indicated pH. Scale bars = 0.5 cm. ±SD, n = 3. Sample pairs were analyzed using the independent t-test. Asterisks denote statistical significance determined using the t-test as follows: $^{*}p < 0.05$, $^{**}p < 0.01$, $^{***}p < 0.001$, $^{****}p < 0.0001$.

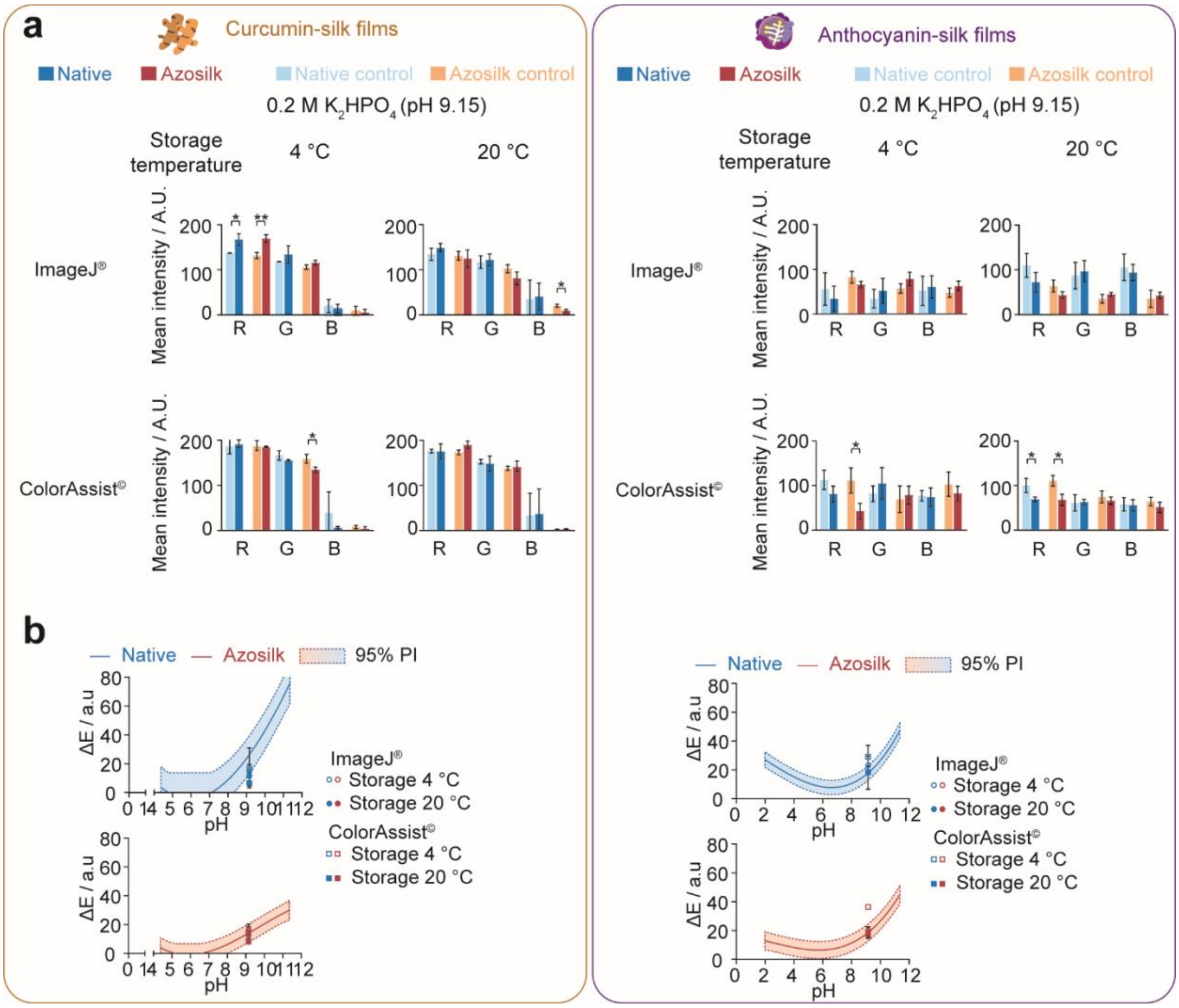

Figure S11. The colorimetric stability of curcumin and anthocyanin-loaded native silk and azosilk medium thickness films containing 0.1% (w/w) iron oxide particles. Following 31 days of storage *in vacuo* at 4 or 20 °C, the color changes after exposure to 0.2 M potassium phosphate buffer (pH 9.15) were monitored in **(a)** the RGB color space using the mean pixel channel intensities, and **(b)** in the Lab color space to calculate the color change (ΔE) of silk films. Digital image colorimetry was undertaken using image pre-processing and analysis in ImageJ® or directly in ColorAssist Lite©. ± SD, *n* = 3. Sample pairs were analyzed using the independent *t*-test. Asterisks denote statistical significance determined using the *t*-test as follows: *p < 0.05, **p < 0.01, ***p < 0.001, ****p < 0.0001. ±SD, *n* = 3.

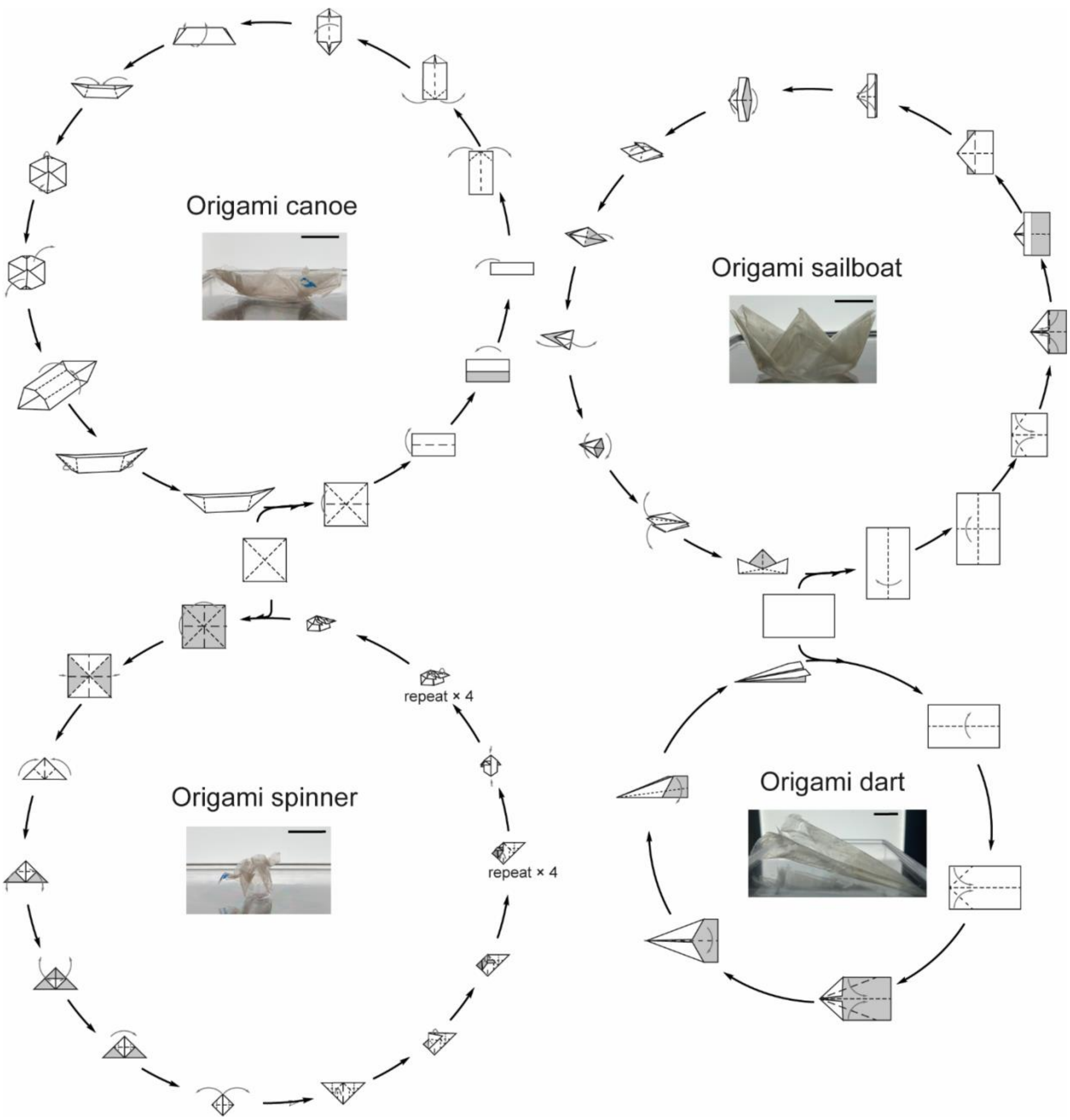

Figure S12. Origami folding and the resulting silk film origami structures.

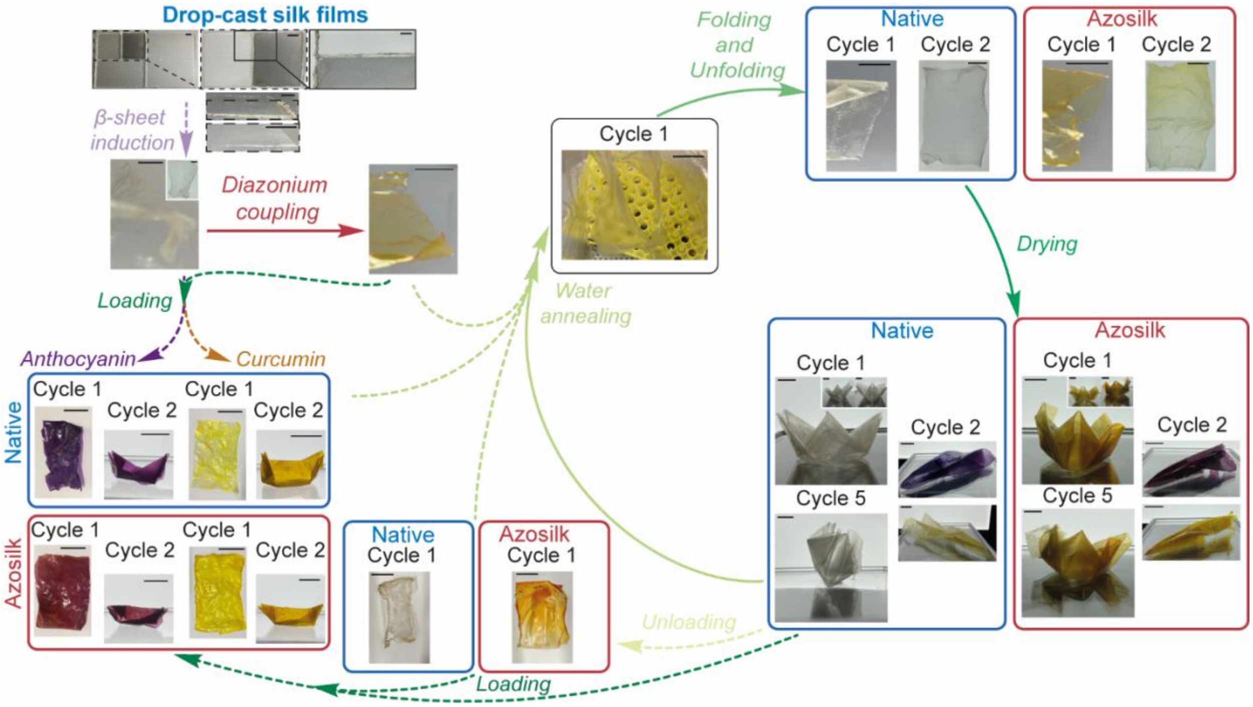

Figure S13. The full workflow for the lab-scale production of smart silk origami eco-sensors.

References

(1) Zhang, J.; Huang, X.; Zou, X.; Shi, J.; Zhai, X.; Liu, L.; Li, Z.; Holmes, M.; Gong, Y.; Povey, M.; Xiao, J. A Visual Indicator Based on Curcumin with High Stability for Monitoring the Freshness of Freshwater Shrimp, Macrobrachium Rosenbergii. *J. Food Eng.* **2021**, *292*, 110290. DOI:10.1016/j.jfoodeng.2020.110290.

(2) Yang, H.; Yang, S.; Kong, J.; Dong, A.; Yu, S. Obtaining Information about Protein Secondary Structures in Aqueous Solution Using Fourier Transform IR Spectroscopy. *Nat. Protoc.* **2015**, *10*, 382–396. DOI:10.1038/nprot.2015.024.

(3) Shimanovich, U.; Ruggeri, F. S.; De Genst, E.; Adamcik, J.; Barros, T. P.; Porter, D.; Müller, T.; Mezzenga, R.; Dobson, C. M.; Vollrath, F.; Holland, C.; Knowles, T. P. J. Silk Micrococoons for Protein Stabilisation and Molecular Encapsulation. *Nat. Commun.* **2017**,

8, 15902. DOI:10.1038/ncomms15902.

(4) Hu, X.; Kaplan, D.; Cebe, P. Determining Beta-Sheet Crystallinity in Fibrous Proteins by Thermal Analysis and Infrared Spectroscopy. *Macromolecules* **2006**, *39*, 6161–6170. DOI:10.1021/ma0610109.

(5) Tomar, S. Converting Video Formats with FFmpeg. *Linux Journal* **2006**, *146*, 10.

(6) Chen, H. zhi; Zhang, M.; Bhandari, B.; Yang, C. hui. Novel PH-Sensitive Films Containing Curcumin and Anthocyanins to Monitor Fish Freshness. *Food Hydrocolloids* **2020**, *100*, 105438. DOI:10.1016/j.foodhyd.2019.105438.

(7) Mary, C. P. V.; Vijayakumar, S.; Shankar, R. Metal Chelating Ability and Antioxidant Properties of Curcumin-Metal Complexes – A DFT Approach. *Journal of Molecular Graphics and Modelling* **2018**, *79*, 1–14. DOI:10.1016/j.jmgm.2017.10.022.

(8) Fenger, J. A.; Moloney, M.; Robbins, R. J.; Collins, T. M.; Dangles, O. The Influence of Acylation, Metal Binding and Natural Antioxidants on the Thermal Stability of Red Cabbage Anthocyanins in Neutral Solution. *Food Funct.* **2019**, *10*, 6740–6751. DOI:10.1039/c9fo01884k.

(9) Fedenko, V. S.; Shemet, S. A.; Landi, M. UV–Vis Spectroscopy and Colorimetric Models for Detecting Anthocyanin-Metal Complexes in Plants: An Overview of in Vitro and in Vivo Techniques. *Journal of Plant Physiology* **2017**, *212*, 13–28. DOI:10.1016/j.jplph.2017.02.001.
